# Care pathway analysis and evidence gaps in adult-onset Still’s disease: interviews with experts from the UK, France, Italy, and Germany

**DOI:** 10.3389/fmed.2023.1257413

**Published:** 2023-09-12

**Authors:** Francesco Ursini, Emily Gregg, Viviam Canon-Garcia, Hilde Rabijns, Katrin Toennessen, Kaz Bartlett, Sara Graziadio

**Affiliations:** ^1^Medicine and Rheumatology Unit, IRCCS Istituto Ortopedico Rizzoli, Bologna, Italy; ^2^Department of Biomedical and Neuromotor Sciences, Alma Mater Studiorum University of Bologna, Bologna, Italy; ^3^York Health Economics Consortium Ltd., York, United Kingdom; ^4^Novartis AG, Basel, Switzerland; ^5^Novartis NV/SA, Vilvoorde, Belgium; ^6^Novartis GmbH, Nuremberg, Germany

**Keywords:** care pathway analysis, adult-onset Still’s disease, qualitative research, research recommendation, evidence gap, clinical practice

## Abstract

**Introduction:**

Adult-onset Still’s disease (AOSD) is a rare systemic inflammatory disease of unknown etiology. Published AOSD data are limited, and clinical guidelines were lacking until recently. Managing AOSD remains largely empirical with uncertainties and high variability about the optimal care pathway. Therefore, we used a qualitative approach to collect clinical judgments from the UK, Italy, France and Germany to inform the development of an agreed care pathway. Our work aimed to decrease the uncertainty associated with clinical practice, inform future research in AOSD, and help identify standardized definitions and outcomes in this population.

**Methods:**

Semi-structured interviews and thematic analysis were conducted. Eleven clinicians were interviewed between May and July 2022: four were based in Italy, three in the UK, two in France, and two in Germany.

**Results:**

In this work, we identified the structure of the typical care pathway for AOSD patients, which can be used to inform future economic models in AOSD. The general structure of the pathway was similar across countries. Non-steroidal anti-inflammatory drugs are prescribed during the diagnostic workup while an additive approach is commonly used in confirmed cases: corticosteroids, conventional synthetic disease-modifying antirheumatic drugs, then biologic disease-modifying antirheumatic drugs (bDMARDs) (dose increased before switching). For severe presentations, more aggressive approaches with higher doses and early use of bDMARDs are used. The main elements of variation among countries and clinicians were the criteria used for diagnosis; order of bDMARDs and preferential treatments for articular and systemic patients; and tests for patient monitoring. There is also a lack of standardized outcome measures making comparisons and evidence synthesis challenging.

**Conclusion:**

We identified important evidence gaps for clinical practice, e.g., reliable tests or scores predictive of disease progression and treatment outcome, and recommendations for research, e.g., reporting of compliance rates and use of the Yamaguchi criteria for clinical study inclusion. Consensus is needed around the use of the Systemic score in clinical practice and the clinical utility of this score. A standardized definition of remission is also required in AOSD, and further research should look to identify and validate the specific laboratory markers to be considered when assessing remission.

## 1. Introduction

Adult-onset Still’s disease (AOSD) is a rare systemic inflammatory disease of unknown etiology (1, 2). AOSD has a heterogeneous clinical presentation which increases the need for more tailored therapeutic strategies to improve long-term outcomes (3). As is often the case for orphan conditions, AOSD data are limited and guidelines were lacking until recently (4). In 2022, some new German guidelines for the diagnosis and treatment of AOSD were published (5).

When there are no (or limited) guidelines to inform the diagnosis and management of a disease, the care pathway both within and among countries is often variable, and treatments are mainly prescribed based on published literature, economic considerations (in some countries), and clinical judgment/experience. Consequently, managing AOSD remains largely empirical (6) with uncertainties and high variability about the optimal care pathway, which could lead to suboptimal outcomes for patients.

Care pathway analysis (CPA) is a useful methodology to identify medical decisions and outcomes in the current care pathway for a specific disease. This method can also be used to identify evidence gaps and unmet clinical needs, especially in situations where there is a paucity of data (7). CPA has been used in diagnostics research [e.g., (8–10)] and has applicability in the context of rare diseases, like AOSD. CPA facilitates the development of a visual representation of the journey made by patients through the healthcare system for a specific disease (i.e., a flow diagram of the care pathway). This flow diagram can be used to inform the structure of an economic model (11) as well as clinical practice and disease management. It is particularly valuable when national or international guidelines for the diagnosis and management of the disease are not available or not followed in clinical practice.

Therefore, we conducted a CPA of the diagnosis and treatment of AOSD, using a methodology which is novel in this field. We used a qualitative approach to collect clinical judgments from different European countries to inform the development of an agreed care pathway. Our work aimed to decrease the uncertainty associated with clinical practice that could be translated into the structure of an economic model for AOSD. A second aim was to inform clinicians about clinical practice and its variability to support their daily decision making about patient management. Clinical experience is particularly important in the context of limited guidelines and published evidence. In addition, this CPA aimed to inform future research in AOSD (both clinical studies and systematic literature reviews) and help identify standardized definitions and outcomes in this population.

Thus, the objectives of our CPA were:

• To understand the care pathway for the identification and treatment of AOSD in the UK and the main differences with other European pathways.
• To explore the order of treatments for AOSD and the rules around treatment switching and discontinuation.
• To determine important clinical outcomes in AOSD.
• To identify evidence gaps and inform areas for future research.

## 2. Materials and methods

### 2.1. Research design

A qualitative evaluation using semi-structured interviews and thematic analysis was conducted. Overall, 11 clinicians with expertise in the diagnosis and management of AOSD from the UK, Germany, Italy and France were interviewed. Confidentiality and anonymity were maintained when conducting, analyzing and reporting this research. Ethical approval was obtained from the Health Sciences Research Governance Committee at the University of York (reference: HSRGC/2022/508/C). The CPA research questions and methods were pre-defined in a protocol (Supplementary Section 1).

To minimize bias and align with best practice guidelines for qualitative studies (12), the research was blinded; the clinicians did not know that the research was funded by Novartis, and Novartis does not know the identity of the interviewees. If requested by the interviewees, this information was disclosed at the end of the project.

The project was guided by the Standards for Reporting Qualitative Research guidelines to ensure rigor and comprehensive reporting (13) (Supplementary Section 6). It was also aligned with recommendations from the British Healthcare Business Intelligence Association (14) and European Pharmaceutical Market Research Association (15).

### 2.2. Eligibility criteria, recruitment, and consent

Clinicians were invited to participate based on their expertise in the disease area, including rheumatologists, immunologists and internal medicine specialists. Clinicians were sought from the UK, Germany, Italy and France and had to be fluent in English because the output from this work was developed from the recorded interviews.

Potential interviewees were sent an email invitation and information sheet (Supplementary Section 2) outlining the purpose of the project. Those who agreed to participate completed a written consent form, including consent for publication (Supplementary Section 2). Overall, 40 clinicians were contacted during recruitment, 11 accepted to be interviewed, thus the response rate was 28%. The interviewees received an honorarium through York Health Economics Consortium for participating in an interview equal to the fair market value in each country.

### 2.3. Topic guide

A topic guide (Supplementary Section 3) was prepared for the interviews and included a draft care pathway flow diagram and a list of interview questions. The care pathway showed the clinical decisions around diagnosis and treatment of AOSD in the UK and was developed based on published pathways within the literature (2, 4, 16). The draft pathway was focused on the UK pathway to inform the development of a UK-based economic model (which will be presented in a separate publication). The draft care pathway was shared with the clinicians before the interviews. The interview questions included those about the clinician’s role and experience; the current care pathway and treatments for AOSD; unmet clinical needs and variations in clinical practice; and outcomes of interest in this population. Where appropriate, additional prompts were included alongside the interview questions to gain a deeper understanding of the questions asked.

The topic guide was piloted by two medical affairs experts working at Novartis. Feedback from the pilot interviews was incorporated into the final version of the topic guide. Data from the pilot interviews were excluded from the final analysis and results.

### 2.4. Interview structure

The interviews were conducted via Zoom (Zoom Video Communications, Inc.) and were scheduled for 60 min between May and July 2022. Two trained researchers (EG and SG), with extensive experience in qualitative research and CPA, conducted the interviews. One researcher led the interview (EG) whilst the other took notes (SG). The researchers were trained to minimize researcher, confirmation and sampling biases during the interviews (17).

The interview audio was recorded and stored in Zoom. The audio recording and interview notes were used to create a summary of the interview. Full transcripts were not produced. The clinicians checked their interview summary for accuracy before the analysis was conducted.

### 2.5. Thematic analysis, synthesis, and care pathway validation

Using an iterative process, the initial draft pathway was refined and updated to reflect clinical practice based on feedback from the interviews.

Thematic analysis [where common themes across the interviews are extracted from the transcriptions (18)] and a qualitative synthesis of the themes and sub-themes were conducted by one researcher (EG) and validated by the second researcher (SG). Data were extracted from the interview summaries into an Excel spreadsheet. Anonymized quotes were provided to illustrate the themes and provide evidence of the main points of interest (12). After finalization of the analysis, a sample of interviewees (5/11) validated the structure of the revised care pathway diagram.

## 3. Results

Of the 11 interviewed clinicians (Supplementary Table 1), four were based in Italy, three in the UK, two in France, and two in Germany. The clinicians were rheumatologists and/or clinical immunologists, experienced in the diagnosis and management of AOSD and had a median experience of 10 years (interquartile range: 6.5–15 years). The results of the thematic analysis, divided into three major themes, are synthesized in this section.

### 3.1. Narrative summary of the care pathway

**FIGURE 1:**
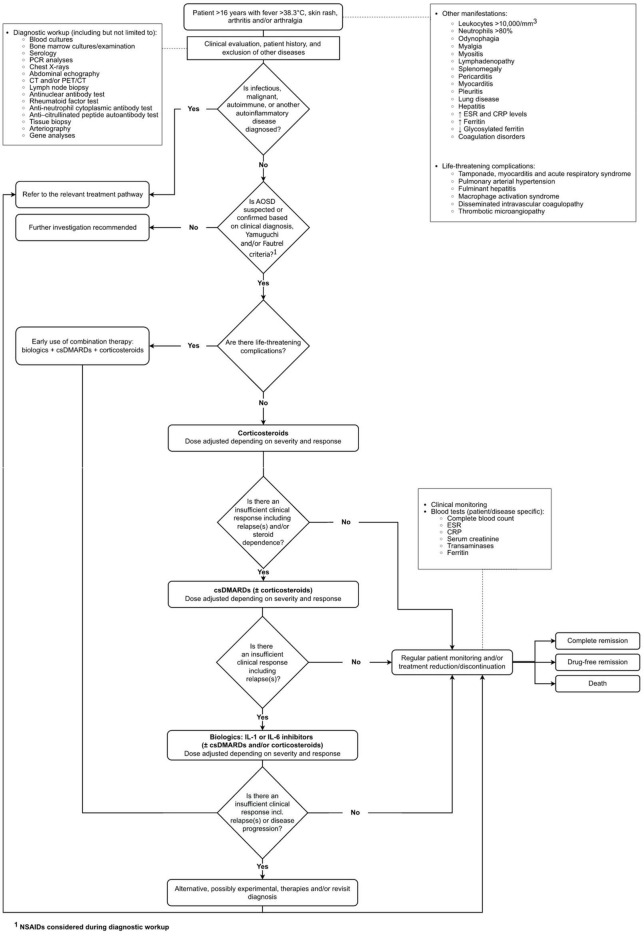
Final validated care pathway flow diagram for AOSD.

The interviewees mostly agreed with the structure of the initial draft pathway (Supplementary Section 3). The final validated care pathway is presented in Figure 1.

In the pathway, the population (patients >16 years) can present with fever, skin rash, arthritis and/or arthralgia. Although these are the cardinal symptoms of AOSD, the clinical presentation is heterogeneous and non-specific.

“I’ve seen patients present with fever and isolated pneumonitis, myocarditis, hepatitis and macrophage activation syndrome (MAS). There are very heterogeneous presentations. It may not just be the four symptoms presented in the pathway (fever, skin rash, arthritis and arthralgia), but they are most common” UK clinician.

There is also an ongoing discussion around the age of disease onset. When discussing this uncertainty, a clinician described a “gray zone between 16 and 18 years.”

The first stage of the pathway involves a clinical evaluation, including a review of patient history and the exclusion of other diseases, such as infectious, malignant or autoimmune diseases or other autoinflammatory diseases. The clinical diagnosis is often based on the Yamaguchi (19) and/or Fautrel criteria (20). While most clinicians agreed with these criteria, some said they are not used in clinical practice and robust clinical criteria are used instead, especially given the heterogeneity of the disease presentation. Some clinicians mentioned that these criteria are for classification not diagnosis, so they are commonly used in clinical trials more than in practice. Also, the Fautrel criteria require the assessment of glycosylated ferritin that is not routinely available in all hospitals, especially in Italy. During the diagnostic workup, non-steroidal anti-inflammatory drugs (NSAIDs) are commonly prescribed.

It was apparent from the interviews that the presence of life-threatening complications (e.g., those in an intensive care unit with MAS or pulmonary involvement) occurs in around 10% to 15% of patients, confirming recent clinical studies (2, 21), and strongly influences treatment choice in AOSD. If the patient has life-threatening complications, the clinician prescribes a combination of biologic disease-modifying antirheumatic drugs (bDMARDs), conventional synthetic disease-modifying antirheumatic drugs (csDMARDs) and corticosteroids early in the disease and uses an aggressive approach to treatment (e.g., high doses and early use of bDMARDs). A small number of clinicians reported that anakinra might be used earlier in the pathway because of its short half-life. The half-life of a drug is related to the time taken for elimination of the drug from the body; it determines the frequency of dosing without influencing the speed of action of the drug.

If there are no life-threatening complications, the first-line treatment is corticosteroids. Some clinicians mentioned that steroids are cheap, effective and work quickly. The complications of steroid use in AOSD are well known and align with those in other diseases, e.g., osteoporosis, hypertension, infections and diabetes (22). There was a general consensus that NSAIDs are not often used in combination with corticosteroids as the first-line treatment.

“After AOSD is diagnosed, I would stop NSAIDs and would move to corticosteroids. I wouldn’t combine NSAIDs and corticosteroids” Italian clinician.

Two clinicians noted that NSAIDs only induce remission in 10–20% of patients, but one added that NSAIDs could possibly be used as the first-line treatment in patients with non-severe disease where corticosteroids could or should be avoided.

A csDMARD (most commonly methotrexate or cyclosporine), or in severe cases a combination of two csDMARDs, are added as the second-line treatment in the first weeks after diagnosis, or earlier if there is an insufficient clinical response to corticosteroids. Generally, the clinicians try to discontinue steroids quickly and do not like to prescribe high doses for long periods to avoid side effects and steroid dependence. In the NHS in England, two csDMARDs must be tried before considering bDMARDs, unless there are issues with tolerability (23).

If there is an insufficient response to the second-line treatment or the patient experiences significant relapses, bDMARDs are introduced as the third-line treatment.

The most commonly used bDMARDs in the UK are interleukin (IL)-1 (anakinra) and IL-6 (tocilizumab) inhibitors (24). In addition to anakinra, another IL-1 inhibitor, canakinumab, is frequently used in Italy, France and Germany but is not approved for AOSD in the UK. Tocilizumab is also prescribed to treat AOSD in Italy and France (despite the lack of regulatory approval from the European Medicines Agency) but is used infrequently in Germany. Further details about approvals for anakinra, tocilizumab and canakinumab are presented in Supplementary Section 4.

There is high variability in the order of bDMARDs used. If there is an inadequate response to the first bDMARD, the clinician adjusts the dose and may consider switching to an alternative bDMARD, but only as a final option due to the limited treatment options available. After IL-1 and IL-6 inhibitors, the interviewed clinicians in Europe (not routinely in the UK) may consider tumor necrosis factor (TNF)-alpha inhibitors and/or Janus kinase (JAK) inhibitors. Use of TNF inhibitors in AOSD was reported in the literature since 2001 and mainly before the availability of IL-1 and IL-6 inhibitors (25). Use of JAK inhibitors is more recent in AOSD (26), and some clinicians noted there are limited data available on their efficacy and safety in this population.

Some AOSD patients experience several relapses, and others maintain remission without any relapses (27). A change in treatment may be considered if the patient experiences relapses. There was agreement among the clinicians that the number of relapses needed before switching treatment depends on the type and intensity of the relapse, efficacy of treatment, the severity of the disease, and patient wishes. If the trigger of the relapse can be identified, for example a viral infection, COVID vaccination or poor compliance, then treatment would not be switched. Furthermore, there is no standardized definition of relapse or guidelines about when to stop or switch treatment in AOSD which is contributing to variation in the care pathway.

Patients with AOSD are followed up by a clinical team, potentially for their whole life in those with chronic disease. The frequency of patient monitoring depends on the stage of the disease and severity. It is rare that patients do not respond to any treatments, but if this happens, then alternative, possibly experimental, therapies will be considered, and patients might be referred to clinical trials. The diagnosis may also be revisited, and some clinicians may have national discussions with other experts in the field. Bone marrow transplants may be considered for AOSD in the UK, but this is exceptional and would be a last resort; these are not considered for AOSD in France, Italy, and Germany.

### 3.2. Variation in the care pathway among countries

#### 3.2.1. Treatment reduction and discontinuation

Treatment reduction or discontinuation is considered when a patient is in remission. The majority of clinicians would slowly taper the corticosteroid first, then discontinue the csDMARD (if the patient is receiving this in combination with bDMARDs), and finally taper the bDMARD (by reducing the dose and increasing the time between the injections). However, the approach depends on the patient and clinical presentation, and some clinicians commented on the lack of guidelines and recommendations for treatment reduction and discontinuation.

The clinicians noted that treatment discontinuation is a very slow process that can take several years, especially if the patient has a severe initial presentation. Some clinicians added that discontinuing the bDMARD can be challenging.

“For those on anakinra, we try to reduce the dose after 2–3 years. First, we reduce to 6 days per week, then after another year, we reduce to 5 days per week and so on. This is a practical approach, but I don’t have any literature to support this” Italian clinician.

Regarding steroid discontinuation, the majority of clinicians agreed that steroids will be discontinued when the patient is in remission. The minority suggested that some AOSD patients may be on long-term low-dose corticosteroids, and another proposed that a steroid dosage of <5 mg/day is considered reasonably safe. The clinicians may also prescribe a short course of steroids to control a mild relapse but not for long-term use.

“Sometimes people relapse if they have an infection. If they have been on the treatment for long time and the disease was well controlled, I would give additional steroids to treat the relapse to regain control” UK clinician.

The time to steroid discontinuation varied between clinicians and depends on the disease. The answers provided ranged from 6 weeks after treatment initiation to a few months after remission.

“A total of 60% of patients can stop steroids if they are treated well at the beginning of their disease. Time to stop steroids depends on the patient. If the clinical conditions allow, steroids should be reduced every 2 weeks, and time to discontinuation depends on the initial dose” Italian clinician.

#### 3.2.2. Split into articular and systemic AOSD and preferential bDMARDs

Previous studies reported a split between two phenotypes of AOSD (systemic and articular) when discussing the treatment pathway and management of the disease (28, 29). This dichotomy is based on the different cytokine profile between articular and systemic AOSD. It has been suggested in the literature that systemic manifestations respond better to IL-1 inhibitors, such as anakinra or canakinumab, and articular manifestations respond better to IL-6 inhibitors, such as tocilizumab (30).

The majority of the clinicians interviewed disagreed with these suggestions and reported that the patient response to bDMARDs does not seem to depend on the patient being in the articular or systemic phase. Thus, these clinicians prescribe anakinra or canakinumab first followed by tocilizumab, regardless of phenotype. This is in accordance with recent evidence from the AutoInflammatory Diseases Alliance (AIDA) registry showing good outcomes in both systemic and chronic articular patients with canakinumab as the first-line bDMARD (31). On the other hand, four clinicians suggested that they tend to prescribe different bDMARDs for articular and systemic patients; however, it is important to note that one of these clinicians only manages systemic patients. For systemic patients, these clinicians prescribe one or two IL-1 inhibitors (anakinra or canakinumab) before tocilizumab. For articular patients, most prioritize tocilizumab (followed by anakinra or canakinumab). Generally, if a clinician reported using the same bDMARDs for all patients, they disagreed with the split in phenotype between articular and systemic AOSD. In contrast, those clinicians who reported using preferential bDMARDs tended to support the split. This suggests that the split could be useful for AOSD research, when exploring outcomes for different groups of patients, but it does not usually inform treatment decisions in clinical practice (31).

#### 3.2.3. Regular monitoring in AOSD

The frequency of patient monitoring varies and depends on the patient’s treatment or clinical presentation. Five clinicians discussed how monitoring changes over time.

“At the beginning (I see them) every week, then every month, then every 3 months, and finally every 6 months, (with a) progressive spacing of visits” Italian clinician.

If the patient is in remission or drug-free remission, the majority of clinicians agreed they are seen every 6–12 months. The other clinicians proposed more regular monitoring every 3–4 months. This variability might be country specific more than linked to the specific disease, with German clinicians monitoring chronic diseases more often.

Several tests are used during monitoring which are patient and disease specific. While the tests presented in the pathway represent the core tests used across countries (Figure 1), several additional tests are also considered. Because the variability is very high, we summarized the information in Table 1.

**TABLE 1:** Tests used during monitoring.

**UK**
• Coagulation tests are only for patients with an acute illness, MAS, or for patients at risk of complications and not for routine monitoring. • One clinician does not measure ferritin routinely. • Another clinician does not measure ESR during monitoring but will check liver and renal function.
**Italy**
• One clinician uses troponin, NT-proBNP and creatine kinase to assess cardiac function in those with myocarditis. • One clinician includes lipids and electrolytes. • Another includes urine cultures, fibrinogen serum, and the Systemic or modified Systemic score to assess disease activity: “Fibrinogen serum level is useful with ESR because if both are elevated it shows a true inflammatory cause, otherwise ESR can be high for many reasons (e.g., anemia or high cholesterol serum levels).”
**France**
• One clinician does not use ESR during monitoring, but includes full liver tests, not only transaminases but also alkaline phosphatase and gamma-glutamyl transferase, as well as fasting glucose (if on steroids) and potassium levels.
**Germany**
• One clinician measures serum amyloid, MRP8/14 biomarkers, and IL-18 during routine monitoring. • Another clinician measures IL-18 and S100 proteins like calprotectin during routine monitoring. Some patients have lung involvement and may need imaging in addition to the clinical and laboratory measures.

ESR, erythrocyte sedimentation rate; IL-18, interleukin-18; MAS, macrophage activation syndrome; MRP, myeloid-related protein; NT-proBNP, N-terminal pro B-type natriuretic peptide; UK, United Kingdom.

#### 3.2.4. Proportion of patients on each treatment

Overall, there was variability between clinicians regarding the proportion of patients on treatment and those in drug-free remission (Table 2). There was some uncertainty in the values provided, and these estimations were based on clinical opinion rather than data. The variability in response is likely due to the different treatment pathways and systems for AOSD across the countries as well as the range in disease severity and presentation assessed by the clinicians.

**TABLE 2:** Proportion of patients on different treatments.

csDMARDs	bDMARDs				Drug-free remission
	Anakinra	Canakinumab*	Tocilizumab	Other bDMARDs	
**UK**					
∼30% split between csDMARDs and drug-free remission	∼35%		∼35%		∼30% split between csDMARDs and drug-free remission
∼50%	∼35%		∼5%		∼10%
30–40% with low-dose steroids	15–20%		15–20%		∼30%
**Italy**					
∼20% on methotrexate and steroids or steroids only	∼40%	∼30%	∼10%		0%**
3–5 patients (4% to 7%)	35 patients (∼47%)	23 patients (∼31%)	10 patients (∼13%)		2 patients (∼3%)
40–45% with corticosteroids	∼10%	∼10%	∼10% (IL-6 inhibitors rather than tocilizumab specifically)	TNF inhibitors: <5%	25–30%
50–55%	∼20%	∼10%	∼10%		10–15%
**France**					
∼15%	∼20%	∼10%	∼15%	TNF and JAK inhibitors: <10%	∼25%
**Germany**					
	45–55%	40–50%			<5%
	∼40%	∼40%			∼20%

bDMARD, biologic disease modifying antirheumatic drug; csDMARD, conventional synthetic disease-modifying antirheumatic drug; IL-6, interleukin-6; JAK, Janus kinase; TNF, tumor necrosis factor. It was outside the scope of this project to estimate the level of uncertainty in the estimations provided. Most clinicians reported percentages for each treatment, but C4 reported the number of patients on each treatment.

*Canakinumab is not currently used routinely in the UK for reimbursement reasons. **Mostly treats those with chronic or relapsing forms of AOSD rather than monophasic patients.

There was high variability in the proportion of patients prescribed bDMARDs, especially in Italy where this ranged between 28 and 91% of patients. The proportion of patients in drug-free remission also varied among and within countries, but it was always ≤30% (see Table 2).

#### 3.2.5. Variation in dose across treatments

The dose of each treatment reported by the clinicians is shown in Supplementary Table 2. Two UK and one French clinician suggested that treatment dose is consistent across patients.

“(Dose) is linked to the molecule. We follow the recommendation by the manufacturers” French clinician.

The other clinicians suggested that treatment dose depends on the patient and severity of the disease, any side effects, and the prescribing clinician. There is less variation in dose for bDMARDs, especially for tocilizumab.

### 3.3. Areas of uncertainty in AOSD

#### 3.3.1. Predictors of disease progression or response

The clinicians agreed that in AOSD there are no validated biomarkers predictive of disease progression or response to treatment, and “this is a problem for AOSD.” A range of potential predictors were mentioned (Table 3).

**TABLE 3:** Predictors of disease progression or response.

**UK**
• Mortality is high in patients with MAS. • The initial response to steroids is important and influences how quickly bDMARDs are prescribed.
**Italy**
• The severity of the manifestations will influence the course of AOSD and outcomes. • Ferritin is a good predictor of aggressive disease in the short and long term. Patients with important articular disease are more likely to have a chronic disease. • Ferritin is associated with the occurrence of complications, and CRP the occurrence of complications and mortality. • CRP could be associated with articular erosions and progression to a chronic articular phenotype but is not validated on a large scale.
**Germany**
• A high Systemic or Still’s activity score is associated with MAS. • Lung involvement could predict a negative long-term outcome.

AOSD, adult-onset Still’s disease; bDMARD, biologic disease modifying antirheumatic drug; CRP, C-reactive protein; MAS, macrophage activation syndrome; UK, United Kingdom.

#### 3.3.2. Definition of remission (partial and complete)

Whilst remission (both complete and drug-free) is an important outcome in the AOSD pathway, clinical studies and literature reviews, a standardized definition is still lacking. However, we are aware that while preparing this publication the European Alliance of Associations for Rheumatology (EULAR) task force is working on this, with an ongoing initiative to develop a standardized definition of remission and a disease activity score for AOSD: “CLI113–Development and validation of a EULAR disease activity score in AOSD: the “DAVID” project” (32, 33). Results from this EULAR project should be available in the near future.

“I always have this discussion with colleagues. Clinical and lab remission differ depending on which biomarkers you use. There are no clear recommendations about how to define remission in AOSD, so it always depends on the position of the clinician. That is a problem” German clinician.

There was a general consensus that complete remission is the absence of clinical symptoms and the normalization of at least one laboratory marker, with most clinicians suggesting that several laboratory markers should be considered. Important laboratory markers to be considered included: cell numbers (platelets, white blood cells), neutrophil counts, S100 proteins (S100A12), C-reactive protein (CRP), erythrocyte sedimentation rate (ESR), ferritin, leukocytosis with neutrophilia, serum amyloid, and IL-18. Clinicians noted that the definition should also include the corticosteroid dose (in addition to the clinical and laboratory features), and a target dosage of ≤5 mg/day for complete remission was proposed.

For partial remission, there was more variation in the definitions provided. Two UK and two Italian clinicians stated that this concept is not helpful in clinical practice or research and suggested that partial remission is just “active disease” or “low disease activity.”

The other definitions included the absence of clinical symptoms with abnormal laboratory markers or vice versa; clinical and laboratory remission with a medium dose of steroids (between 5 mg and 10 mg/day); or the persistence of some clinical features and some abnormal laboratory markers. One German clinician proposed partial remission could be an improvement of >75% in the main symptoms, as used in rheumatoid arthritis.

#### 3.3.3. Variation in remission length (treatment vs. patient’s disease)

The majority of clinicians were unable to comment on whether the variation in remission length is due to treatment or the patient’s disease because no evidence is available. This is likely the reason for the large variability in responses around the topic.

Three clinicians proposed that the variation is due to patient and disease characteristics with a drug retention rate of ∼60% for all treatments, thus no treatment is superior.

On the other hand, two clinicians suggested the variation is due to treatment. One said that bDMARDs induce more sustained remission than csDMARDs. The other noted that IL-1 inhibitors induce the greatest percentage of and the most sustained remission, if administered early during the disease course. Finally, two Italian clinicians suggested the variation is due to a combination of the patient’s disease and the treatment they received.

#### 3.3.4. Treatment compliance and monitoring

There was disagreement among the interviewed clinicians about treatment compliance in AOSD because compliance data are lacking in this population. Five clinicians suggested that treatment non-compliance is rare, but the others implied that compliance differs between treatments. One UK clinician explained that treatment non-compliance is a major problem in rheumatology in general, but it is possibly better in AOSD because of its severity.

The four Italian clinicians suggested that compliance is worse for anakinra because of the daily injections, with pain and discomfort at the injection site experienced by some patients. The clinicians reported that around 15–20% of patients experienced pain at the injection site, and 10–20% changed bDMARDs because of injection site reactions. One Italian clinician noted that there is a poor compliance for some patients, but it depends on the number of drugs administered. They added that “40% of patients do not comply with bDMARDs with a short half-life” (with more frequent doses).

“Anakinra is a problem because of the daily injections. Often, they want the lowest possible number of injections or tablets. Some patients are not compliant with methotrexate, so they might stop taking it without telling us. With canakinumab, compliance is ok. With steroids the patient may reduce the amount they are taking because of the side effects” Italian clinician.

In general, only two clinicians provided a guestimate for non-compliance in this population and answers ranged between 10 and 20%. Six of the clinicians suggested that compliance to daily doses may decrease over time, but the other five clinicians did not support this.

“I think (compliance to daily doses) does change over time, especially in patients who don’t like needles. This is definitely an advantage of canakinumab over anakinra, but I don’t know how much of a problem it is” UK clinician.

There was high uncertainty about how often insufficient compliance triggers flares, with several of the interviewed clinicians unable to quantify this. There was a wide range of other estimates provided, which ranged from no connection to 90% correlation between the two events.

#### 3.3.5. Important outcomes, clinical measures of severity (systemic score) and patient-reported outcome measures

Overall, there is a lack of standardized outcome measures in AOSD, with a broad range used throughout research and clinical practice (33) (see further details in Supplementary Table 3). Remission outcomes, including clinical, laboratory and drug-free remission, were the most commonly reported outcomes to measure efficacy and effectiveness. Partial remission and time to reach remission were also offered. Several clinicians mentioned outcomes linked to steroid use, including steroid dose in remission, ability to stop steroids, time to stop steroids, and steroid-sparing effects, and more general treatment use, including dose reductions and drug retention.

Some important safety outcomes included rates of complications, survival rate after MAS, and mortality after MAS. Infections, injection site reactions, and corticosteroid complications (e.g., toxicity, diabetes, skin problems, osteoporosis and psychiatric issues) were offered as the most clinically relevant treatment-related adverse events in AOSD.

The Systemic score (34) is a measure of disease severity that can be clinically used to predict poor patient outcomes and life-threatening evolution of disease (mortality, MAS and lung disease). At the time of diagnosis, patients scoring a cut-off of 7 on the Systemic score have been identified as high risk of AOSD-related death (35–37). Despite this, use varies between clinicians. Only five clinicians use the Systemic score [or modified versions of the score (38, 39)] in clinical practice, with one of these only using it in combination with steroid dose. Whereas some other clinicians use clinical evaluation rather than a score to assess disease severity.

“Pouchot (Systemic score) is most used and most relevant, but there is no consensus” French clinician.

Two clinicians suggested that the utility of the Systemic score is limited.

“The Pouchot (Systemic) score can only be measured at the beginning of the disease when looking at all different domains of the patient. You can’t repeat it in remission or during a flare so you can’t compare the initial score with a later timepoint. This is a real problem–it is not for daily use” German clinician.

In terms of other measures of severity, the Disease Activity Score 28 (40) or American College of Rheumatology criteria (41) are sometimes used for articular patients, but these don’t take into account systemic features. Some clinicians mentioned the Still’s activity score, myeloid-related protein 8/14 biomarker, HScore (developed for reactive hemophagocytic syndrome), and MAS score in systemic juvenile idiopathic arthritis (SJIA); however, these may not have been validated in AOSD. As mentioned in Section “3.3.2. Definition of remission (partial and complete),” a EULAR task force is currently developing and validating a disease activity score for AOSD (32, 33).

The interviewed clinicians agreed that there are no AOSD-specific patient-reported outcome (PRO) measures. The most frequently mentioned generic tools were the 36-Item Short Form Survey (SF-36) (42) and Health Assessment Questionnaire (HAQ) (43).

#### 3.3.6. Generalizability of SJIA to AOSD

There is an ongoing debate in the scientific community around whether SJIA and AOSD lie on a continuum of disease given the similar clinical presentation, cytokine profiles and gene expression patterns (44, 45). The majority of the interviewed clinicians agreed that some SJIA data can be generalized to AOSD, although three clinicians did not support this (see further details in Supplementary Section 5). This is important for future evidence generation in AOSD; for example, when sourcing inputs for economic models given the rarity of the disease and that AOSD-specific data are still lacking.

“The extrapolation of data from SJIA to AOSD has been consistent, but it is possible there could be a difference for some aspects. For now, SJIA is a good model because of the limited data for AOSD” French clinician.

## 4. Discussion

There are uncertainties and high variability regarding the optimal treatment pathway in AOSD. In this CPA, we identified the structure of the typical care pathway for AOSD patients, which is applicable to clinical practice in the UK, Italy, Germany and France. Whilst the general structure of the care pathway is representative, there is some variation in clinical practice among countries and clinicians at various stages, which are reported as evidence gaps in Section “4.1. Strengths and limitations of the CPA.” While preparing this publication there was a second ongoing EULAR initiative aimed at providing a set of recommendations for diagnosis and management of AOSD: “QoC011–EULAR/Pediatric Rheumatology European Society (PRES) recommendation for the diagnosis and management of SJIA and AOSD” (46). New recommendations from this initiative were presented recently at the EULAR 2023 Annual Meeting. However, at the time of preparing this manuscript, these were unpublished. When available, the recommendations will help to standardize definitions, disease assessment, and treatment approaches in this population. They will also help to address some of the evidence gaps identified in this work and will decrease the uncertainty and variability associated with clinical practice in AOSD.

This CPA demonstrated that there is also a lack of standardized outcome measures, with a wide range used throughout research and clinical practice, making comparisons and synthesis of evidence difficult. In rare diseases patient recruitment is time consuming and conducting clinical studies is very expensive, thus the evidence base is usually limited to a small number of studies. To build a strong evidence base, which is essential when evaluating new therapies and diagnostic tests, these studies should be aggregated. Using standardized definitions and outcomes would allow for robust aggregation of single studies, increasing the interpretability and generalizability of results and accelerating the introduction of new therapies into practice, which are particularly needed in AOSD. Recommendations for future research in this population are presented in Section “4.2. Evidence gaps for clinical practice.”

### 4.1. Strengths and limitations of the CPA

This work aligned with best practice guidelines for qualitative research (12, 14, 15). The hierarchy of evidence places expert opinion at the bottom of the pyramid (i.e., the lowest quality of evidence) suggesting these data are less reliable. However, expert opinion can provide useful information in certain situations. For example, in rare diseases like AOSD (47) where there is a lack of high-quality data, and it is difficult and expensive to conduct large trials. No formal elicitation of quantities was included in the interviews which is a potential limitation of this study. Further studies should be conducted to supplement the results presented in this manuscript and to explore the key themes highlighted in more depth.

Another limitation of this CPA is that a small sample size (*n* = 11) was included. In part, this is representative of the limited number of AOSD experts in Europe. However, a larger sample may have provided further insights and richer data. Although only 11 experts participated, these were from the UK, France, Italy, and Germany. The experts were also recruited from different research and clinical centers to cover different perspectives on the disease management across the countries of interest. Given the CPA was focused on four European countries, this might limit its global applicability; this limitation should be considered in future research. Furthermore, no patients (or patient representatives) were recruited to take part in this CPA. This represents another limitation that should be addressed in further studies in this population. Capturing the patients’ perspective could provide valuable insights into treatment experiences and outcomes in AOSD, especially given the rarity of the disease. A final strength is that this research was blinded. The clinicians did not know the sponsor of the research which minimized response bias throughout the interviews.

### 4.2. Evidence gaps for clinical practice

• Treatment reduction and discontinuation should be considered when a patient is in remission. In practice, the majority of the interviewed clinicians seem to slowly taper the corticosteroid first, then discontinue the csDMARD (if the patient is receiving this in combination with bDMARDs), and finally taper the bDMARD. Future guidelines should provide recommendations about the order of discontinuation; the best approach for tapering each treatment; the convenience of combining csDMARDs and bDMARDs; and the suggested time to discontinuation. When available, we recommend that guidance from EULAR and PRES should be used to inform treatment approaches (46).
• Some elements related to the order of treatments were common among clinicians. NSAIDs are prescribed during the diagnostic workup while an additive approach is commonly used in confirmed cases of AOSD: corticosteroids, csDMARDs, then bDMARDs (dose increased before switching). For severe presentations, more aggressive approaches with higher doses and early use of bDMARDs are used. However, further evidence is needed to establish the optimal treatment strategy with bDMARDs and if there are preferential bDMARDs for articular and systemic AOSD. In addition, clear criteria about when to add or switch treatments, including the recommended time points for assessment and decision, are needed.
• Compliance data for all treatments are lacking in this population. Future work should investigate treatment compliance in AOSD patients (possibly in registries too) and potential strategies to improve this in clinical practice (for example, regular compliance monitoring and assessment for patients on bDMARDs). This is important information that might be related to flares; therefore, investigations in this topic are particularly recommended. An initial step could be the reporting of compliance data in all prospective clinical studies and trials.
• There are no AOSD-specific PRO measures. SF-36 and HAQ are sometimes used, but they are not specific to this population. If developed and validated, the PRO measures could be useful to support treatment decisions and the evaluation of new therapies.
• Consensus is needed around the use of the Systemic score in clinical practice and the clinical utility of this score. Monitoring tools that are specifically developed for clinical practice and can be used at different time points in the disease are also needed.

### 4.3. Recommendations for research

• The overall structure of the validated care pathway (Figure 1) should be used to inform future economic evaluations of treatments in AOSD and will help to reduce the uncertainty around the structure of economic models in this population.
• The Yamaguchi criteria (19) are recommended for inclusion of patients in clinical studies, more so than the Fautrel criteria (20) that require the assessment of glycosylated ferritin which is not always available. Standardized criteria are preferred over unstructured clinical criteria in situations of clinical uncertainty like in AOSD. The broad use of the same criteria in clinical research would facilitate data aggregation and meta-analysis.
• The split in phenotype between articular and systemic AOSD could be useful in research studies when exploring outcomes for different groups of patients, even though the split does not seem to reflect clinical practice.
• A standardized definition of remission is lacking in AOSD. We suggest the use of drug-free remission in clinical trials (all AOSD-related drugs), and complete remission defined as the resolution of clinical signs and symptoms and the normalization of some laboratory markers. Further research is needed to identify and validate the specific laboratory markers to be considered when assessing remission. When exploring this, cell numbers (platelets, white blood cells), neutrophil counts, S100 proteins, CRP, ESR, ferritin, leukocytosis with neutrophilia, serum amyloid, and IL-18 should be considered. The usefulness and a definition of partial remission is still to be established. When available, we recommend the EULAR definition of remission is used in future research in AOSD (32, 33).
• Alongside remission, other important outcomes for clinical studies assessing the efficacy of treatments include steroid dose in remission, ability to stop steroids, time to stop steroids, steroid-sparing effects, treatment dose reductions, and drug retention. Important safety outcomes include rates of complications, survival rate after MAS, and mortality after MAS. Clinically relevant treatment-related adverse events include infections, injection site reactions, and corticosteroid complications. These outcomes should be considered in the design of clinical studies and systematic reviews. When available, the EULAR disease activity score for AOSD should be used in future research (32, 33).

• In situations where AOSD data are lacking, e.g., when sourcing inputs for economic models, we suggest that data could be generalized from SJIA if considered appropriate. When using data from SJIA, a rationale for the generalization should be provided.

## Data availability statement

The datasets presented in this article are not readily available because the participants of this study did not give written consent for their data to be shared publicly, so the authors are not allowed to share the supporting data (i.e., the transcripts of the interviews). Requests to access the datasets should be directed to sara.graziadio@york.ac.uk.

## Ethics statement

The studies involving humans were approved by the Health Sciences Research Governance Committee at the University of York (reference: HSRGC/2022/508/C). The studies were conducted in accordance with the local legislation and institutional requirements. The participants provided their written informed consent to participate in this study.

## Author contributions

FU: Supervision, Validation, Writing—review and editing. EG: Data curation, Formal analysis, Investigation, Visualization, Writing—original draft. VC-G: Conceptualization, Funding acquisition, Writing—review and editing. HR: Writing—review and editing. KT: Writing—review and editing. KB: Project administration, Visualization, Writing—review and editing. SG: Conceptualization, Funding acquisition, Investigation, Methodology, Resources, Supervision, Writing—review and editing.
