# Supplementary material for: Care pathway analysis and evidence gaps in adult-onset Still’s disease: interviews with experts from the UK, France, Italy, and Germany
Source: Front Med (Lausanne). 2023 Sep 12;10:1257413. doi: 10.3389/fmed.2023.1257413 (PMC10523327; doi:10.3389/fmed.2023.1257413)
Supplement: Supplementary file 1 [file Data_Sheet_1.PDF]

Supplementary Material

1 CPA Protocol

1.1 Background

Adult-onset Still's disease (AOSD) is a rare systemic inflammatory disease of unknown etiology [1, 2]. The main symptoms are fever, joint pain, and skin rashes [3, 4]. Some patients only experience one episode of the disease (monocyclic AOSD), whilst others experience multiple episodes separated by periods of remission (polycyclic or intermittent AOSD), and some people have chronic/persistent AOSD where symptoms are continuous [5]. Monocyclic, polycyclic and chronic/persistent are the phenotypes of disease. Each case of the disease is one of these types, and patients do not progress from one to the other. These labels can only be assigned to patients retrospectively, after assessing their pattern of disease.

Patients with active AOSD often have lower health-related quality of life (HRQoL) compared with patients in remission [6]. Furthermore, AOSD is associated with life-threatening complications, including macrophage activation syndrome (MAS), pulmonary arterial hypertension, and thrombotic thrombocytopenic purpura (TTP) [7]. The lack of a specific serologic biomarker, non-specific clinical presentation, and the rarity of AOSD often result in a substantial delay in diagnosis, which is compounded by the paucity of clinical guidelines [8].

Current evidence supports that pro-inflammatory cytokines, including interleukin (IL)-1, IL-6, IL-17, IL-18, tumor necrosis factor (TNF)-α, and interferon (INF)-γ, are closely associated with various clinical manifestations of AOSD [9-11].

Like AOSD, systemic juvenile idiopathic arthritis (SJIA) is a systemic inflammatory disease characterized by fever, joint pain and skin rash [12] but occurs in children under the age of 16 [13]. It is generally accepted that SJIA and AOSD represent a disease continuum with different ages of onset, known together as Still's disease [10, 14, 15]. The European Medicines Agency (EMA) and Scottish Medicines Consortium (SMC) have accepted evidence that the outcomes from trials in the SJIA population can be generalized to AOSD [16, 17].

The work proposed herein is limited to AOSD only. Two different phases of AOSD have been described; a systemic/inflammatory phase (characterized by acute onset, fever, evanescent rash, weight loss and other systemic manifestations) and the articular/arthritis predominant phase (characterized by indolent onset and systems mainly affecting the joints) [18, 19]. Within the systemic phase, the disease may be monocyclic or polycyclic (intermittent) whereas the articular predominant phase tends to be chronic [20]. The phases of the disease are defined based on what the dominant manifestations are at that time. Therefore, patients can progress from systemic to articular over time. This project includes patients with all phases of AOSD.

1.2 AOSD Treatments

Corticosteroids (particularly glucocorticoids) are commonly used as first-line treatment for AOSD once a diagnosis has been made, and disease modifying anti-rheumatic drugs (DMARDs) such as methotrexate are often considered in combination with glucocorticoids [8, 21, 22]. The initial response to glucocorticoids is generally good, although even high-dose glucocorticoids sometimes fail to induce remission [23]. However, glucocorticoid toxicity causes iatrogenic problems. Decreasing glucocorticoids frequently induces flares and relapse of symptoms, which necessitates increases in dose, organ damage and long-term side effects, such as osteoporosis, metabolic disease, and increased risk of cardiovascular disease [23, 24]. Methotrexate is frequently used in AOSD for its steroid-sparing effect. If methotrexate fails to control the disease, other conventional DMARDs (e.g. azathioprine, tacrolimus) may be used. When corticosteroids or conventional DMARDs do not effectively control AOSD, biologic agents should be used [25].

Given the activation of IL and TNF in AOSD, anti-cytokine biologic disease modifying anti rheumatic drugs (bDMARDs) such as inhibitors of TNF, IL-1, or IL-6 have been used to treat patients who are refractory to conventional treatment and patients who have systemic complications [20, 26-29]. Biologic DMARDs offer a more target-specific mechanism of action than conventional DMARDs and have emerged as an important therapeutic alternative in patients with Still’s disease of all ages.

The IL-6 inhibitor tocilizumab is approved (among other indications) for treatment of SJIA in the US and the European Union (EU) for children aged one year and older, with active SJIA, who have not had an adequate response to non-steroidal anti-inflammatory drugs (NSAIDs) or corticosteroids. Tocilizumab can be used on its own or in combination with methotrexate [22, 30]. It is not approved specifically in AOSD in the EU or US [31, 32].

The IL-1 receptor antagonist (IL-1Ra) anakinra, which blocks both IL-1α and IL-1β biologic activity, has been approved (among other indications) for SJIA in Australia and the USA, and for both SJIA and AOSD by the EMA [33, 34]. Anakinra can be given as monotherapy or in combination with other anti-inflammatory drugs and DMARDs [35].

Canakinumab (Novartis) is a recombinant human monoclonal antibody which inhibits IL-1β [2, 36]. Clinical trial results for the treatment of AOSD with canakinumab have been promising [37]. However, there is a limited amount of clinical trial data for this drug and indication. Despite this, canakinumab is now recommended in the British National Formulary (BNF) for the treatment of AOSD by subcutaneous injection once every four weeks [36]. Canakinumab can be used alone or in combination with methotrexate [38].

1.3 AOSD Care Pathway

The care pathway for the diagnosis and treatment of AOSD varies between areas [21], owing to a lack of national and international guidelines [3]. The pathway to AOSD diagnosis in the UK is particularly unclear and variable across patients, meaning there is frequently a delay in treatment [21]. Once the diagnosis is confirmed, the order of the treatments is also variable and strongly dependent on clinical judgement.

1.4 Project Aims

A systematic review of biologic treatments for AOSD was conducted by YHEC for Novartis in 2021 [39]. Novartis has now commissioned an economic model to assist in decision making regarding the position of canakinumab in the treatment pathway, as well as two systematic reviews (a clinical and economic review) to source the inputs for the model.

Considering the variability in the AOSD care pathway, the economic model would benefit from an analysis of the current UK care pathway (care pathway analysis, CPA) to decrease the uncertainty associated with its structure. The CPA can also inform the outcomes of interest for the systematic reviews. Although the care pathway will be developed from the UK perspective, clinical opinion will also be captured from other countries (namely Germany, Italy and France) which will provide further insight for the model and reviews.

Due to the limited number of randomised control trials (RCTs) in this clinical area, the systematic reviews will include evidence derived mainly from real world evaluations (e.g. retrospective, non-randomized studies). This evidence can be weak and difficult to interpret, thus we will convene a panel of experts to validate the model inputs obtained from the reviews and address any input gaps. This expert opinion elicitation should improve the robustness of the economic model outputs.

Therefore, the aims of this project are:

- To deliver a CPA of the diagnosis and treatment of AOSD.
- To conduct an expert opinion elicitation to validate and integrate the economic model inputs.

This protocol describes the methods and timeline for the interviews with clinicians which will form part of the CPA and will be completed in phase one of this project. The expert opinion elicitation will be completed in phase two and will be discussed in more detail with Novartis at a later date.

1.5 Methods

1.5.1 Research Design

A qualitative evaluation using semi-structured interviews and thematic analysis will be conducted in this CPA. Researchers from YHEC will interview healthcare professionals (HCPs) based in the UK, Germany, Italy or France who are involved in the diagnosis and management of AOSD.

Overall, 12 interviews will be conducted. YHEC will organise and conduct the interviews and will also be responsible for all the legal and contracting duties relating to the interviews.

The research will be blinded; the HCPs will not know that the research is being conducted by Novartis. If requested by the HCP, this information will be disclosed at the end of the research project. In all research materials for the HCPs, Novartis will be referred to as a ‘pharmaceutical company’.

This project will be aligned with the British Healthcare Business Intelligence Association (BHBIA) and European Pharmaceutical Market Research Association (EphMRA) guidelines.

1.5.2 Research Questions

The following research questions will be addressed throughout this work:

- What is the care pathway for the identification and treatment of patients with AOSD in the UK? What are the main differences with other European pathways for patients with AOSD?
- What is the order of treatments? What are the "rules" around treatment switching and discontinuation?
- From a clinical perspective, what are the clinical outcomes of interest to judge efficacy and effectiveness of treatment?
- Which safety and HRQoL outcomes are clinically relevant?
- Can data from SJIA be generalised to AOSD?

The topic guide will include specific research questions around the broad themes mentioned above.

1.5.3 Ethical Approval

Ethical approval is not required for this CPA. However, as publication of the results is of interest to Novartis, we advise that ethical approval is obtained.

If the project timeline and progress align with the ethics committee submission dates, ethical approval will be obtained from the Health Sciences Research Governance Committee at the University of York.

1.5.4 Selection of Interviewees

Overall, 12 HCPs will be recruited for this project using purposive sampling. HCPs will be selected based on their expertise in the disease area, including rheumatologists, immunologists, and internal medicine specialists with experience in the diagnosis and treatment of AOSD. HCPs will be sought from the UK, Germany, Italy, and France.

The HCPs should be independent from Novartis to minimise bias and increase robustness of the methodology.

Whilst Novartis will identify the potential HCPs, YHEC will solely conduct all engagement and communication. Novartis will compile a long list of potential HCPs that may be eligible based on their clinical experience and expertise in AOSD. This longlist will help mitigate the risk of not identifying the agreed number of HCPs within the timescales of the project. The identity of the HCPs who participate in the project will not be shared with Novartis.

1.5.5 Interviewee Recruitment and Consent

Following written approval from the Novartis Patient Oriented Program (POP) team, potential interviewees will be sent an email invitation (see attached email invitation) and information sheet (see attached information sheet and consent form) outlining the purpose of the project and the adverse event reporting requirements. They will also be offered the chance to ask any questions.

Those who agree to take part in the interview will be asked to sign and return a written consent form (see attached information sheet and consent form) stating that they: are happy to participate; consent to the interview being recorded; consent for anonymised quotes to be included in the project report to illustrate the themes and provide evidence of the main points; and are happy for any relevant quotes related to adverse events to be shared with Novartis (as per the adverse event reporting requirements). The HCP will also be asked whether their name and contact details can be shared with

Novartis if any adverse events are reported during the interview (if not, the relevant quotes will be kept anonymous, except for in France where sharing their contact details with any relevant adverse events is a requirement for participation) and if YHEC can contact them after the interview regarding phase two of this project.

The interviewees may withdraw from this research at any stage of the project. However, if this is after data analysis has been completed, their data will still be included in the study.

YHEC will arrange a suitable date and time with the interviewee and will send a meeting request in advance. The interview will be scheduled for 60 minutes. YHEC will also send a reminder email before the interview and will ask the interviewee to confirm attendance.

Interviewees will be informed that they should not disclose any personal information (apart from when asked about their role and experience) or confidential patient information during the interview. If there is any disclosure of confidential information, this will not be transcribed and will be deleted from the audio recording.

1.5.6 Interviewee Honorarium

The HCPs will receive an honorarium for participating in an interview. The exact honorarium for each country will be proposed by YHEC based on previous projects and is subject to agreement with Novartis.

1.5.7 Eligibility Criteria

To be eligible for an interview, the HCPs must have relevant experience of the diagnosis and management of AOSD. The HCPs must be based in the UK, Germany, Italy, or France. Additionally, the interviewees must be fluent in English as the output from this work will be developed from the recorded interviews.

1.5.8 Sample Size

Due to the exploratory nature of this work, we have opted to interview a small and carefully selected group of HCPs to provide the relevant clinical picture. The maximum sample size for the interviews is 12. This sample size is based on reaching information saturation and is estimated from similar qualitative studies [40-42].

1.5.9 Topic Guide

A topic guide will be prepared before the interviews. This will include:

- A care pathway flow diagram showing the clinical decisions around diagnosis and treatment in the UK. This care pathway will be developed based on published pathways within the literature (for example [2-4]) and expert advice from within Novartis. The draft pathway will be agreed with Novartis before the interviews are conducted.
- A list of interview questions. This will include questions about the HCP's role and experience, the current care pathway and treatments for AOSD to inform the model structure, and the outcomes of interest for the systematic reviews. Where appropriate, additional prompts will be included alongside the interview questions to gain a deeper understanding of the questions asked.

Before the interviews are conducted, the topic guide will be piloted on two experts from the Novartis medical team. Feedback from these pilot interviews will be incorporated into the final draft. These pilot interviews will not be included in the final analysis or results.

1.5.10 Interview Structure

The interviews will be conducted via Zoom (Zoom Video Communications, Inc.). Two trained researchers, with experience in CPA and qualitative research methods, will conduct the interviews; one will lead the interview whilst the other takes notes.

Minimising potential bias is a key consideration in qualitative research. Common sources of bias include researcher, confirmation and sampling bias [45]. The researchers are trained to minimise these biases during the interview.

The draft care pathway (together with the information sheet and consent form) will be sent to the HCPs before the interviews. This will be discussed during the interview to capture any missing elements or inaccuracies in the proposed pathway. The pathway will be used as a starting point to discuss the unmet clinical need and variations in practice for the treatment of AOSD.

The interview audio will be recorded and stored in Zoom. This will be automatically deleted from Zoom after 180 days. YHEC will download and save a copy of this recording which will be used to create a transcript of the interview and to check understanding of key points. The transcript will be produced using the transcribe feature in Office 365 and/or the live transcript function in Zoom.

A summary of the key points from each transcript will be produced. The HCPs will be given the opportunity to check this summary for accuracy. This will be shared via the University of York DropOff Service.

Novartis will not be provided with the interview recordings or transcriptions. If required, the summary of each interview can be provided via email or the University of York DropOff Service.

1.5.11 Thematic Analysis and Synthesis

Thematic analysis (i.e. common themes across the interviews are extracted from the transcriptions) will be conducted by one researcher whilst the other will validate the extraction though additional checking. Data will be extracted into a spreadsheet. Thematic analysis is a flexible method which allows themes to emerge from the data collected [43] and is commonly used in CPA (for example [43]).

Once themes are extracted and agreed, the researcher will conduct the qualitative synthesis of the themes and will summarise them into the report. Anonymised quotes will be provided to illustrate the themes and provide evidence of the main points of interest.

1.5.12 Reporting

Following the analysis, the researchers will communicate the results of interest to the YHEC reviewers and modellers as required. The results of interest to the reviewers are likely to include the relevant clinical and safety outcomes, interventions and comparators, time limits, and population terms. The results of interest to the modellers are likely to include the care pathway flow diagram and

key model inputs (e.g. number of relapses allowed before treatment switching and impact of previous relapse on the probability of future relapses).

A CPA report will be provided. The report will include the methods used to conduct and analyse the interviews; the synthesis of the interview analysis; and the final care pathway description and flow diagram. If any major pathway variations across the UK and/or Europe are identified throughout the interviews, these will be flagged in the care pathway description. The report will also include a short description of the methodology used for the expert opinion elicitation, a summary of the workshop discussion, and a table with the input values elicited by the experts (see Section 2. 14).

To ensure rigour and comprehensive reporting, the content and format of the CPA report will be guided by the Standards for Reporting Qualitative Research (SRQR) guidelines [44].

1.5.13 Adverse Event Reporting

If any adverse events / product complaints associated with a Novartis product are mentioned during the interviews, YHEC will report these to the Novartis Patient Safety team using the Patient Pharmacovigilance Intake tool. YHEC will provide Novartis with the relevant quotes from the transcript within 24 hours from when the adverse event was reported. Novartis will not be provided with the full interview transcript or any recordings. YHEC will comply with the Novartis adverse event reconciliation requirements.

1.5.14 Quality Assurance

Quality assurance will be applied at all stages of this project, including protocol, topic guide and care pathway development; conducting the interviews; thematic analysis and synthesis of the interviews; and report writing, with two researchers involved in all phases of this work. Each draft of the report will be quality assured, and all deliverables (protocol, topic guide and final report) will be signed off by a member of the senior management team.

Each member of the project team has successfully completed the following training:

- Novartis Adverse Event Reporting (modules A and B).
- BHBIA Legal and Ethical Guidelines for Market Researchers.
- BHBIA Adverse Event Reporting in Market Research.
- EphMRA Code of Conduct.
- EphMRA Adverse Event Reporting.

1.5.15 Expert Opinion Elicitation (Phase Two)

Following completion of phase one of this work, a subgroup of the interviewed HCPs (n = 3 to 4) will be invited to participate in a panel to validate the model structure and inputs obtained from the systematic reviews and to elicit any missing inputs [46]. Pragmatic methods, informed by the Sheffield Elicitation Framework (SHELF) [47, 48], will be used for the expert opinion elicitation.

1.5.16 Data Management

Confidentiality and anonymity will be maintained when conducting, analysing, and reporting the interviews.

Interviewees will be assigned a unique code which will be used throughout the project. Data will never be presented with real names. The interviewees' identification data and unique code will be stored in a password protected folder on a password protected computer.

All data (consent forms, interview notes, transcriptions, and recordings) will be stored in a password protected folder on a password protected computer. All data will be anonymised (except for the consent forms). These data files will only be accessible to the YHEC researchers working on this project. One of the researchers involved with this project is based in Italy. This researcher will access all data via YHEC's shared filestore. Novartis will not be provided with the consent forms, transcriptions or recordings, except for any relevant quotes when reporting adverse events. If the HCP does not consent to sharing their name and contact details when reporting any adverse events, the relevant quotes provided to Novartis will be kept anonymous. This does not apply to the HCPs in France, as sharing their contact details with any relevant adverse events is a requirement for participation in this project.

All data will be securely retained for a period of six years and will be disposed of appropriately after this period. The data will be added to the YHEC Data Register which is used to keep a record of datasets containing sensitive and personal data. The register includes how long the data need retaining for, a date by which the data should be deleted, and confirmation that the data have been deleted by the person responsible for this.

1.5.17 CPA Protocol References

1. Efthimiou P, Paik PK, Bielory L. Diagnosis and management of adult onset Still's disease. Ann Rheum Dis. 2006;65:564-72.

2. Giacomelli R, Ruscitti P, Shoenfeld Y. A comprehensive review on adult onset Still's disease. J Autoimmun. 2018;93:24-36.

3. Efthimiou P, Kontzias A, Hur P, Rodha K, Ramakrishna GS, Nakasato P. Adult-onset Still's disease in focus: clinical manifestations, diagnosis, treatment, and unmet needs in the era of targeted therapies. Semin Arthritis Rheum. 2021;51(4):858-74.

4. Eugen Feist, Stéphane Mitrovic, Bruno Fautrel. Mechanisms, biomarkers and targets for adult-onset Still's disease. Nature Reviews. Rheumatology. 2018;14(10):603-18.

5. National Organization for Rare Disorders. Adult-onset Still's disease. NORD; 2021. [cited 28th October 2021]. Available from: https://rarediseases. org/rare-diseases/adult-onset-stills-disease/.

6. Chi H, Jin H, Wang Z, Feng T, Zeng T, Shi H, et al. Anxiety and depression in adult-onset Still's disease patients and associations with health-related quality of life. Clin Rheumatol. 2020;39(12):3723-32.

7. Efthimiou P, Kadavath S, Mehta B. Life-threatening complications of adult-onset Still's disease. Clin Rheumatol. 2014;33(3):305-14.

8. Pak S, Pham C. Delay in the diagnosis of adult-onset Still's disease. Cureus. 2017;9(6):e1321.

9. Kadavath S, Efthimiou P. Adult-onset Still's disease-pathogenesis, clinical manifestations, and new treatment options. Ann Med. 2015;47(1):6-14.

10. Inoue N, Shimizu M, Tsunoda S, Kawano M, Matsumura M, Yachie A. Cytokine profile in adult-onset Still's disease: comparison with systemic juvenile idiopathic arthritis. Clin Immunol. 2016;169:8-13.

11. Jamilloux Y, Gerfaud-Valentin M, Martinon F, Belot A, Henry T, Sève P. Pathogenesis of adult-onset Still's disease: new insights from the juvenile counterpart. Immunol Res. 2015;61(1-2):53-62.

12. Jung JY, Kim JW, Suh CH, Kim HA. Roles of interactions between toll-like receptors and their endogenous ligands in the pathogenesis of systemic juvenile idiopathic arthritis and adult-onset Still's disease. Front Immunol. 2020;11:583513.

13. National Institute for Health and Care Excellence. Systemic juvenile idiopathic arthritis: canakinumab. London: NICE; 2014. Available from: https://www. nice. org. uk/advice/esnm36/resources/systemic-juvenile-idiopathic-arthritis-canakinumab-pdf-1502680927183045.

14. Colafrancesco S, Manara M, Bortoluzzi A, Serban T, Bianchi G, Cantarini L, et al. Management of adult-onset still's disease (AOSD) with IL-1 inhibitors: evidence-and consensus-based statements by a panel of Italian experts. Ann Rheum Dis. 2019;78(Suppl 2):575-76.

15. Nirmala N, Brachat A, Feist E, Blank N, Specker C, Witt M, et al. Gene-expression analysis of adult-onset Still's disease and systemic juvenile idiopathic arthritis is consistent with a continuum of a single disease entity. Pediatr Rheumatol Online J. 2015;13:50.

16. European Medicines Agency. Ilaris - EPAR: assessment report. Amsterdam: European Medicines Agency; 2016. Available from: https://www. ema. europa. eu/en/documents/variation-report/ilaris-h-c-1109-ii-0043-epar-assessment-report-variation_en. pdf.

17. Scottish Medicines Consortium. Anakinra 100mg/0. 67mL solution for injection in prefilled syringe (Kineret). Glasgow: 2018. Available from: https://www. scottishmedicines. org. uk/media/3764/anakinra-kineret-final-sept-2018-amended-021018-for-website. pdf.

18. Maria ATJ, Le Quellec A, Jorgensen C, Touitou I, Riviere S, Guilpain P. Adult onset Still's disease (AOSD) in the era of biologic therapies: dichotomous view for cytokine and clinical expressions. Autoimmun Rev. 2014;13(11):1149-59.

19. Vercruysse F, Barnetche T, Lazaro E, Shipley E, Lifermann F, Balageas A, et al. Adult-onset Still's disease biological treatment strategy may depend on the phenotypic dichotomy. Arthritis Res Ther. 2019;21(1):53.

20. Gerfaud-Valentin M, Jamilloux Y, Iwaz J, Seve P. Adult-onset Still's disease. Autoimmun Rev. 2014;13(7):708-22.

21. Seco T, Cerqueira A, Costa A, Fernandes C, Cotter J. Adult-onset Still's disease: typical presentation, delayed diagnosis. Cureus. 2020;12(6):e8510.

22. NHS England. Clinical commissioning policy: anakinra/tocilizumab for the treatment of adult-onset Still’s disease refractory to second-line therapy (adults) [210801P] (URN: 1609). London: 2021. Available from: https://www. england. nhs. uk/wp-content/uploads/2021/08/1609-Tocilizumab-for-AOSD-Final-August-2021-. pdf.

23. Kaneko Y, Kameda H, Ikeda K, Ishii T, Murakami K, Takamatsu H, et al. Tocilizumab in patients with adult-onset still's disease refractory to glucocorticoid treatment: a randomised, double-blind, placebo-controlled phase III trial. Ann Rheum Dis. 2018;77(12):1720-29.

24. Coutinho AE, Chapman KE. The anti-inflammatory and immunosuppressive effects of glucocorticoids, recent developments and mechanistic insights. Mol Cell Endocrinol. 2011;335(1):2-13.

25. Wang MY, Jia JC, Yang CD, Hu QY. Pathogenesis, disease course, and prognosis of adult-onset Still's disease: an update and review. Chin Med J (Engl). 2019;132(23):2856-64.

26. Al-Homood IA. Biologic treatments for adult-onset Still's disease. Rheumatology (Oxford). 2014;53(1):32-8.

27. Asanuma YF, Mimura T, Tsuboi H, Noma H, Miyoshi F, Yamamoto K, et al. Nationwide epidemiological survey of 169 patients with adult Still's disease in Japan. Mod Rheumatol. 2015;25(3):393-400.

28. Castaneda S, Martinez-Quintanilla D, Martin-Varillas JL, Garcia-Castaneda N, Atienza-Mateo B, Gonzalez-Gay MA. Tocilizumab for the treatment of adult-onset Still's disease. Expert Opin Biol Ther. 2019;19(4):273-86.

29. Castaneda S, Atienza-Mateo B, Martin-Varillas JL, Serra Lopez-Matencio JM, Gonzalez-Gay MA. Anakinra for the treatment of adult-onset Still's disease. Expert Rev Clin Immunol. 2018;14(12):979-92.

30. European Medicines Agency. RoActemra. Amsterdam: European Medicines Agency; Last updated 10 December 2021. [cited 24 February 2022]. Available from: https://www. ema. europa. eu/en/medicines/human/EPAR/roactemra.

31. Food and Drug Administration. Actemra prescribing information. Silver Spring, Maryland: 2021. Available from: https://www. accessdata. fda. gov/drugsatfda_docs/label/2021/125472s044lbl. pdf.

32. Datapharm. RoActemra 162 mg solution for injection in pre-filled syringe. Summary of product characteristics. Surrey, UK: Electronic Medicines Compendium; 2021. [cited 24 February 2022]. Available from: https://www. medicines. org. uk/emc/product/5357/smpc.

33. Food and Drug Administration. Anakinra prescribing information. Maryland: 2015. Available from: https://www. accessdata. fda. gov/drugsatfda_docs/label/2016/103950s5175lbl. pdf.

34. Datapharm. Package leaflet: Kineret 100 mg/0. 67 ml solution for injection in pre-filled syringe. Anakinra. Surrey, UK: 2020. Available from: https://www. medicines. org. uk/emc/files/pil. 559. pdf.

35. European Medicines Agency. Kineret EPAR assessment report. Amsterdam: 2020. Available from: https://www. ema. europa. eu/en/documents/variation-report/kineret-h-c-363-ii-0073-epar-assessment-report-variation_en. pdf.

36. British National Formulary. Canakinumab. NICE; 2021. [cited 29th October 2021]. Available from: https://bnf. nice. org. uk/drug/canakinumab. html.

37. Kedor C, Listing J, Zernicke J, Weiß A, Behrens F, Blank N, et al. Canakinumab for treatment of adult-onset Still's disease to achieve reduction of arthritic manifestation (CONSIDER): phase II, randomised, double-blind, placebo-controlled, multicentre, investigator-initiated trial. Ann Rheum Dis. 2020;79(8):1090-97.

38. European Medicines Agency. Illaris: EPAR - Summary of product characteristics. 2020. Available from: https://www. ema. europa. eu/en/documents/product-information/ilaris-epar-product-information_en. pdf.

39. York Health Economics Consortium. Systematic review on the use of biologics in adult-onset Still’s disease. York: YHEC; 2021.

40. Hicks T, Winter A, Green K, Kierkegaard P, Price DA, Body R, et al. Care pathway and prioritization of rapid testing for COVID-19 in UK hospitals: a qualitative evaluation. BMC Health Serv Res. 2021;21(1):532.

41. Jones WS, Suklan J, Winter A, Green K, Craven T, Bruce A, et al. Diagnosing ventilator-associated pneumonia (VAP) in UK NHS ICUs: the perceived value and role of a novel optical technology. Diagn Progn Res. 2022;6(1):5.

42. Charman S, Okwose N, Maniatopoulos G, Graziadio S, Metzler T, Banks H, et al. Opportunities and challenges of a novel cardiac output response to stress (CORS) test to enhance diagnosis of heart failure in primary care: qualitative study. BMJ Open. 2019;9(4):e028122.

43. Braun V, Clarke V. Using thematic analysis in psychology. Qualitative Research in Psychology. 2008;3(2):77-101.

44. O'Brien BC, Harris IB, Beckman TJ, Reed DA, Cook DA. Standards for reporting qualitative research: a synthesis of recommendations. Acad Med. 2014;89(9):1245-51.

45. Florczak KL. Best available evidence or truth for the moment: bias in research. Nurs Sci Q. 2022;35(1):20-24.

46. Rossi SH, Blick C, Nathan P, Nicol D, Stewart GD, Wilson ECF. Expert elicitation to inform a cost-effectiveness analysis of screening for renal cancer. Value Health. 2019;22(9):P981-87.

47. Oakley JE, O'Hagan A. SHELF: the Sheffield Elicitation Framework (version 4). School of Mathematics and Statistics, University of Sheffield, UK; 2019. Available from: http://tonyohagan. co. uk/shelf/.

48. O'Hagan A, Buck CE, Daneshkhah A, Eiser JR, Garthwaite PH, Jenkinson DJ, et al. Uncertain judgements: eliciting experts' probabilities: John Wiley & Sons, Ltd; 2006.

2 Information Sheet and Consent Form

2.1 Information Sheet

2.1.1 Title

Care Pathway Analysis and Expert Opinion Elicitation for Adult-Onset Still's Disease

2.1.2 Introduction

A large pharmaceutical company has commissioned York Health Economics Consortium (YHEC) to undertake a care pathway analysis (CPA) of the diagnosis and treatment of adult-onset Still's disease (AOSD). The pharmaceutical company has compiled a longlist of potential participants that may be eligible for this research, based on their clinical experience and expertise in AOSD. We are inviting you to take part in an interview (in English) as part of this project. The list of potential participants was compiled from information available in the public domain. If you decide to participate, we will not share your identity with the pharmaceutical company. If you are interested, we will disclose the name of the pharmaceutical company at the end of this research project. Please see some further information about the project below.

2.1.3 Purpose

AOSD is a rare systemic inflammatory disease of unknown aetiology. The main symptoms are fever, joint pain and skin rashes. Patients with active AOSD often have lower HRQoL compared with patients in remission. Furthermore, AOSD is associated with life-threatening complications, for example macrophage activation syndrome. Treatment for AOSD includes non-steroidal anti-inflammatory drugs (NSAIDs), corticosteroids, non-biological disease-modifying antirheumatic drugs (DMARDs) and biological DMARDs.

The care pathway for the diagnosis and treatment of AOSD is unclear and variable across patients, owing to a lack of national and international guidelines. As a result, there is frequently a delay in diagnosis and treatment for AOSD patients.

CPA allows the identification of medical decisions within the current pathway and involves interviews with relevant clinicians. The aim of this project is to undertake a CPA of the diagnosis and treatment of AOSD. This work will inform future economic modelling of treatments for AOSD as well as outcomes of interest for SLRs in this area.

If you decide to participate in this research, you will be asked to sign a consent form. You will receive an honorarium (equal to the fair market value in your country) for participating in an interview, and the contracting will be between YHEC and yourself (or your institution, if required). You will receive payment after the interview. You can withdraw from this research at any time. If you decide to withdraw after data analysis has been completed, your data will still be included in the study.

2.1.4 Interview Structure

You will be asked at attend a videoconference interview which will be scheduled for 60 minutes between May and June 2022. We will organise the interview around your availability. The interview will be conducted and recorded using Zoom. Two researchers from YHEC will conduct the interview; one will lead the interview whilst the other takes notes.

A draft care pathway for AOSD (together with the information sheet and consent form) will be sent to you before the interview and you will be asked to comment on this during the interview. Some personal information will be collected during the interview about your role within the healthcare system and the city or hospital where you work.

We will export and save a copy of the audio recording which will be used to produce a transcript of the interview and to check understanding of key points. The transcript will be produced using the transcribe feature in Word (Office 365) and/or the live-transcript function in Zoom. All the recordings will be anonymised, and your name will not be saved in the recording, transcript, or interview notes. You will not have access to the interview recording or transcript, but we will send you a summary of the interview. You will have the opportunity to check this for accuracy.

During the interview you should not disclose any additional personal information (other than information about your role as mentioned earlier) or any personal information of your patients. If there is any accidental disclosure of confidential information, this will not be transcribed and will be deleted from the audio recording.

Once all the interviews have been conducted, we will complete a thematic analysis (i.e. common themes across the interviews are extracted from the interview notes). Once themes are extracted and agreed, we will conduct a qualitative synthesis of the themes and will summarise them into a project report, which will be sent to the pharmaceutical company. We may include anonymised quotes from your interview in the report to illustrate the themes and provide evidence of the main points of interest. The results of this work may also be disseminated via scientific publications (e.g. conference abstracts/presentations or journal articles). We are planning to obtain ethical approval from the Health Sciences Research Governance Committee at the University of York.

In separate projects, the pharmaceutical company has also commissioned an economic model of treatments for AOSD and two SLRs to source the inputs for the model. In phase two of this CPA project, 3 to 4 of the interviewed HCPs will be invited to participate in a workshop to validate the model structure and inputs.

2.1.5 Adverse Event Reporting

As this research has been commissioned by a pharmaceutical company, we are required to pass on any adverse events / product complaints pertaining to their products that are mentioned during the interview. If this happens, we will need to collect details and report the adverse event, even if you have already reported this. You will be asked whether you consent to YHEC passing on your name and contact details to the company's drug safety department for their follow up, but you may choose to remain anonymous. This will have no impact on the confidentiality and anonymity associated with the interview itself.

2.1.6 Data Protection

You will be assigned a unique code which will be used throughout the project. Data will never be presented with real names.

All data (identification data, consent forms, interview notes, transcriptions and recordings) will be stored in password protected folders on a password protected computer. These data files will only be accessible to the YHEC researchers working on this project. One of the researchers involved with this project is based in Italy. This researcher will access all data via YHEC's shared filestore. The

pharmaceutical company funding this research will not be provided with the consent forms, transcriptions, or recordings, except for any relevant quotes related to adverse events raised during the interview. Unless you consent to YHEC passing on your name and contact details to the company's drug safety department for their follow up, these quotes will be kept anonymous.

A summary of the interview will be shared with you and the pharmaceutical company funding this research (if requested by them) using the University of York DropOff Service.

All data will be securely retained for a period of six years and will be disposed of appropriately after this period. The data will be added to the YHEC Data Register which is used to keep a record of datasets containing sensitive and personal data. The register includes how long the data need retaining for, a date by which the data should be deleted, and confirmation that the data have been deleted by the person responsible for this.

The YHEC privacy policy is available here: https://yhec.co.uk/privacy-policy/. As explained in Section 4 of the privacy policy, you have a number of rights in relation to your personal data. These include the right to:

- Find out how we process your personal data.
- Request that your personal data is corrected if you believe it is incorrect or inaccurate.
- Withdraw your consent to our processing of your personal data.
- Obtain a copy of the personal information that we hold about you. We will take steps to verify your identity before responding to your request and will respond as soon as possible (after receipt of confirmation of your identity) and in any event within one month.
- If you would like to exercise any of your rights outlined in this policy or have any questions about the way in which YHEC handles your personal data, please contact us in writing at yhec@york.ac.uk.

2.1.7 Contact Details

If you have any further questions or require more information, please contact:

Emily Gregg

emily.gregg@york.ac.uk

Sara Graziadio

sara.graziadio@york.ac.uk

If you would like independent advice or to make a complaint, please contact:

Dr Matthew Taylor

matthew.taylor@york.ac.uk

2.1.8 Consent Form

Please complete and return to emily.gregg@york.ac.uk. Please put your initials in the empty boxes if you agree with the following statements.

I confirm that I am happy to participate in an interview.	
I confirm that I am happy for the interview to be recorded.	
I confirm that I am happy for anonymised quotes to be included in the project report and any future scientific publications to illustrate the themes and provide evidence of the main points of interest.	
I understand that if I mention any relevant adverse events / product complaints during the interview, this information will be passed to the pharmaceutical company commissioning this research.	
If I mention any relevant adverse events / product complaints, I consent to my name and contact details being shared with the pharmaceutical company's drug safety department for their follow up.	
I confirm that I am happy for YHEC to contact me after the interview regarding a second phase of this study and other research projects.	
Would you like to know the name of the pharmaceutical company sponsoring this project? If yes, we will inform you at the end of the research project.	Yes / No
**Signature:**	
**Name:**	
**Date:**	

3 Topic Guide (Including Draft Care Pathway Flow Diagram)

3.1 Introduction

- Thank the healthcare professional (HCP) for agreeing to take part.
- Give a brief introduction to the York Health Economics Consortium (YHEC) and its role in the project:
    - YHEC is a health economics consulting company owned by the University of York. YHEC provides national and international consultancy in health economics and outcomes research to the NHS, the pharmaceutical and healthcare industries.
    - YHEC has been commissioned by a large pharmaceutical company to undertake a care pathway analysis (CPA) of the diagnosis and treatment of Adult-Onset Still's Disease (AOSD). This work will inform future economic modelling of treatments for AOSD as well as outcomes of interest for SLRs in this area.
- Check the HCP has signed the consent form and is happy for the interview to be recorded.
- Remind the HCP that the interview is scheduled for 60 minutes.
- Remind the HCP that as explained on the information sheet, the pharmaceutical company commissioning this research must keep records of any side effects or complaints that people may have about their products, and we must assist the company in meeting its legal obligations. Therefore, if, during the interview, there is any reference made to a side effect or complaint about a medicine/medical device, we will let the company know about this even if it has already been reported directly to the company or the regulatory authorities. Explain that if any AEs are reported in this interview, the company may want to contact the HCP directly to ask some follow-up questions:
    - Check the consent form (before the interview) and confirm if the HCP has agreed for their name and contact details to be passed to the company or not. Check they are happy to proceed.
- Remind the HCP that they should not disclose any personal information or confidential patient information during the interview (other than when asked about their role / previous experience). Explain that if there is any accidental disclosure of confidential information, this will not be transcribed and will be deleted from the audio recording. Remind the HCP that their name will also be deleted from the audio recording.
- Explain that we will begin the interview with some questions about their role/experience before going through the care pathway diagram, and in order to prioritise the most important questions, we will start with questions about treatment before asking some general questions about the disease and diagnosis.
- Check if the HCP has any questions before starting the interview.
- Start the recording in Zoom.

3.2 Interview

3.2.1 Background Questions

- Remind the HCP not to mention their name when answering the background questions.

1. What is your role and speciality, and who do you work for?

2. Which country and city do you work in?

3. How many years of experience do you have in this role?

3.2.2 Care Pathway

- Explain that we will now look at the care pathway which shows our understanding of the decisions involved during diagnosis and treatment of AOSD. Clarify that we are looking for feedback on the pathway based on the HCP's experience to help us understand if the order of treatments is correct and if anything is missing from the pathway.

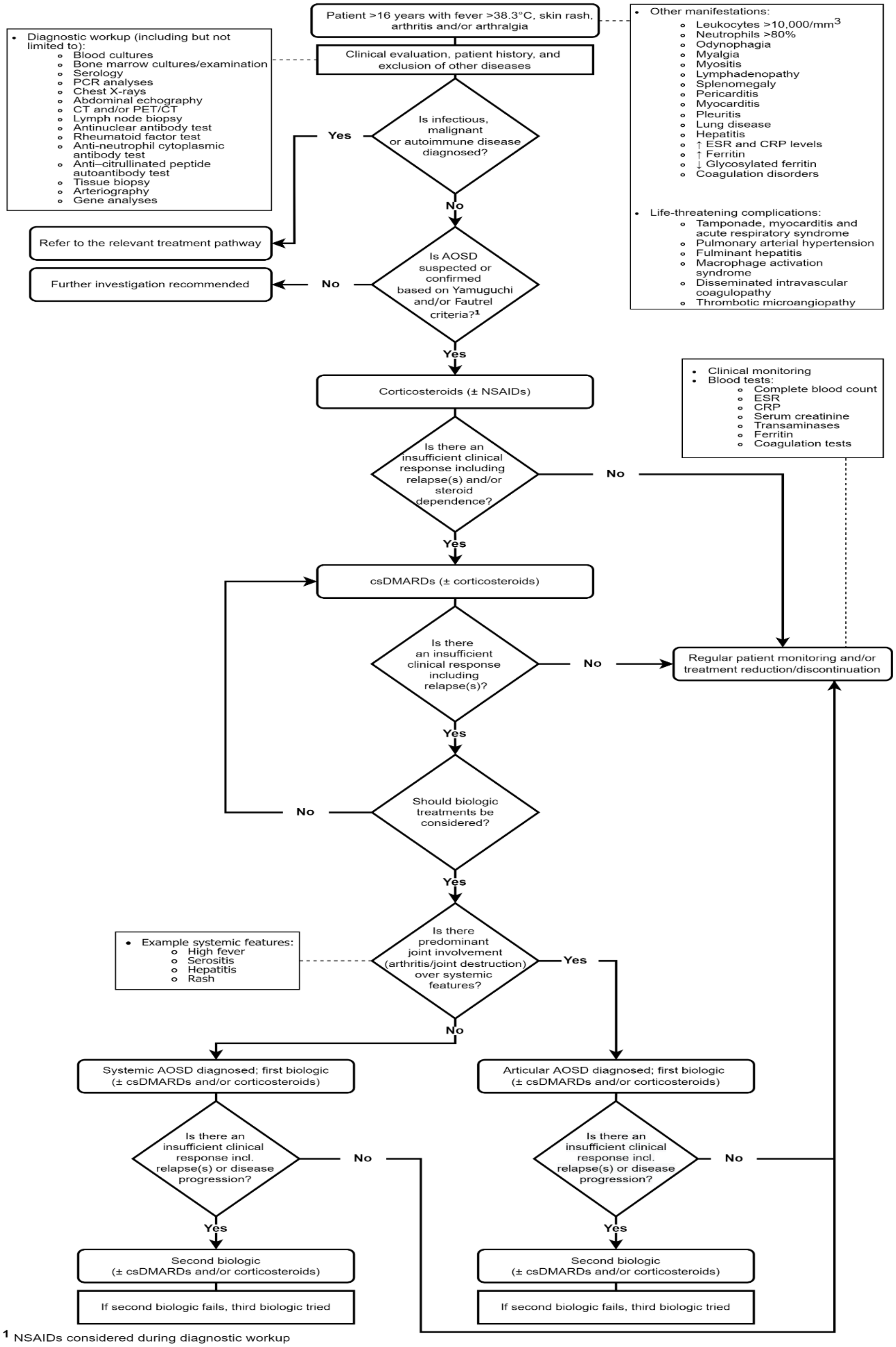

[1] NSAIDs considered during diagnostic workup

3.2.3 Treatment Questions (Country Specific)

1. What are the "rules" around treatment switching after no response or partial response? Do you switch treatment, or do you add new treatments? i.e. is it common for patients to continue to receive their 1st line treatment after commencing 2nd line treatment, and so on?

a. Would you prescribe both csDMARDs and corticosteroids together or just csDMARDs?

b. Are there certain cases where this approach is more or less likely?

c. Would biologics be prescribed with csDMARDs and/or corticosteroids?

d. What happens if the patient does not respond to any biologics? Prompt: Is this common?

2. Are there preferential biologics if a patient is in a systemic phase or articular phase?

3. Which criteria do you follow when reducing or stopping the treatment if remission is achieved (e.g. tapering steroids, stopping csDMARDs, changing the frequency of doses of biologics, or stopping all treatments)?

a. During patient monitoring, are there any specific tests you would use in addition to blood tests?

b. How often are patients monitored?

4. Do you think that the order of treatments could have an impact on clinical outcomes (especially in the long term, like disabilities and disease progression)?

5. Let’s think about switching treatment after relapse. How many relapses would a patient go through on one particular treatment before they would be switched onto a different one?

a. Is this the same going from 1st line to 2nd line and from 2nd line to biologics?

b. If a patient relapses while still taking the treatment that induced a disease remission, are there any cases where they would continue to receive that treatment? Or would it be assumed to not work?

c. Do some treatments tend to induce more sustained remissions or drug free remission than others, or is the variation in remission length mostly down to the patient’s disease rather than the treatment they received? Prompt: Severity, phase of the disease, history of MAS.

d. If a patient relapsed very quickly after stopping a treatment, would you put them back on the same treatment, or would you switch them onto the next treatment line?

e. If the patient returns to the same treatment after relapse or treatment was stopped, would you say there is a decreased probability of response (compared with patients who haven't relapsed after taking said treatment)?

6. What are the most common reasons for switching treatments across the three situations considered until now (no response to treatment, after remission and after relapse)? Prompt: Efficacy, safety, tolerability, compliance and convenience, in this order?

7. What are the determinants of steroid use?

a. Do patients tend to come on and off steroids frequently or is it quite stable?

b. What are the long-term effects of using steroids? Prompt: What are the most common AEs in these patients?

c. When are steroids likely to be discontinued?

8. How much variation is there in dose of a particular treatment administered to a patient (in average across treatments)? Prompt: Does dose fluctuate according to whether or not the patient has active disease, has experienced treatment-related adverse events, and so on?

9. How much of an issue is treatment non-compliance in AOSD?

a. In your opinion, how is compliance in controlled patients to daily doses after 6, 12, and 24 months?

b. How often does insufficient compliance trigger flares?

10. Are bone marrow transplants considered for patients with AOSD (in your country), and if so, which patients would qualify?

3.2.4 General Disease Questions (Not Country Specific)

11. Which complications of AOSD are most clinically relevant (most common and with the highest impact on QoL and mortality)?

12. In addition to the complications discussed above, are there other aspects of the disease or its treatment that have a high impact on patient health-related quality of life (HRQoL) and mortality?

13. What are the clinical measures of systemic severity of AOSD? We are aware of just one, proposed by Pouchot et al. - a score reaching up to 12 points, with a point assigned for each manifestation that is present.

14. We note in some trials, AOSD remission is defined simply as the absence of symptoms of active disease (i.e. body temperature below a certain level, CRP below a cut off, etc.). How would you define remission?

a. Is there such thing as partial remission in AOSD? Prompt: How would you define it?

15. Would you agree that data from SJIA can be generalised to AOSD?

a. Are there any lessons from SJIA that are relevant for AOSD?

b. Are there any aspects of the disease where you would caution against the assumption of generalisability from SJIA?

16. Do you have any thoughts about the societal or community impact of the use of biologics?

3.2.5 Systematic Literature Review Questions (Not Country Specific)

We are planning three SLRs to identify data on clinical effectiveness, resource use and utilities to find data for the economic model. We are now working on the PICO for these reviews.

17. From a clinical perspective, what are the outcomes of interest to judge efficacy and effectiveness of treatment? Prompt: Short- and long-term outcomes.

18. Which treatment-related adverse events are most clinically relevant?

19. What are the commonly used PRO measures (generic or disease specific) to assess QoL in AOSD patients? Prompt: Is the SF-36 commonly used?

20. Where is the greatest resource use and cost burden in this population? Prompt: We are interested in both economic (i.e. financial costs of things) and non-economic (e.g. staff time) data. For example, tests, hospital stay length and frequency, outpatient vs inpatient, primary care.

21. What are the potential confounders in cohort studies or real-world evaluations of Still's disease? i.e. are there any important patient or disease characteristics that might influence the efficacy of the treatment that we might need to consider for the data extraction? For example, is there any clinical or laboratory characteristic that, when present at the first episode of AOSD, could help to predict disease progression (monocyclic to polycyclic / systemic to articular phase) or more generally QoL, mortality or long-term response to biologics? Prompt: Are initial response to steroids or the required dose of steroids predictors?

22. How far back is the literature current/useful for clinical effectiveness, resource use, costs and utilities? Would you recommend some time limits for the SLRs? Which one and how would you justify it (i.e. references)?

3.2.6 Extra Questions (Country Specific)

23. What would you say are the benefits (for either the patient or the healthcare system) of early intervention with biologics, if any?

a. Do you think certain patients would benefit more from this?

b. What about potential harms? Do you think certain patients would be more likely to be harmed by early intervention with biologics?

c. Do you believe there could be a "window of opportunity" in AOSD (i.e. that early use of biologics could reduce the potential of progression from a systemic form to an articular form)?

d. In SJIA some experts are using IL-1 inhibitors as 1st line treatment (with or without steroids). What is your opinion of this approach? Are there any groups of patients with AOSD that could benefit from this approach?

24. Does a history of MAS influence the treatment choice and/or treatment switching?

25. Are certain criteria more commonly used for diagnosis in your country?

a. How long does it typically take to diagnose AOSD in your country?

b. If diagnosis is delayed, what are the implications for the patient?

c. What factors contribute to a delay in diagnosis in your country?

d. How often are patients diagnosed during a MAS episode? Prompt: How often do your patients have a history of MAS during their follow up?

26. Are you aware of any regional differences in diagnosis for AOSD across Europe/in the UK?

27. Is the order of treatments presented in the pathway the same in your country? Prompt: Is anything missing?

28. Do you agree that all patients receive the same 1st line treatment, or does this vary depending on certain characteristics (patient and disease)?

29. Would you prescribe corticosteroids and NSAIDs at the same time?

30. Are there certain cases where this approach is more or less likely?

31. How easy is to identify a patient in the articular phase if not identified during the systemic phase?

32. How would you define refractory AOSD?

33. Are there any delays or bottlenecks in the current pathway in your country? Prompt: What/where are the delays in the pathway?

34. What is the current unmet need in treatments with biologics?

3.2.7 Final Comments

35. Are there any final comments you would like to add?

- Thank the HCP for participating in the project.

Remind the HCP that we will produce a summary of the key points from the interview, and we will send this to check for accuracy.

4 Approvals for biologic disease modifying anti rheumatic drugs (bDMARDs)

The three most commonly used biologic disease modifying anti rheumatic drugs (bDMARDs) are anakinra, canakinumab and tocilizumab. Currently, in the UK, the only biologic DMARD recommended by NICE for the treatment of AOSD is anakinra [1], but NHS England commissioned anakinra and tocilizumab for AOSD [2]. In October 2022, NHS England also recommended canakinumab as a "not for routine commissioning treatment option for adults and children 2 years and over with Still's disease refractory to treatment with (or do not tolerate) anakinra and tocilizumab" [3]. In Europe, canakinumab and anakinra are approved by the European Medicines Agency (EMA) for AOSD [4, 5]. Tocilizumab is approved for treatment of SJIA in the EU for children aged one year and older, with active SJIA, who have not had an adequate response to NSAIDs or corticosteroids. It is not approved specifically in AOSD in the EU or US [6, 7].

4.1 References

1. National Institute for Health and Care Excellence. Anakinra for treating Still's disease. London: NICE; 2020. Available from: https://www.nice.org.uk/guidance/ta685/documents/129.

2. NHS England Specialised Services Clinical Reference Group for Specialised Immunology and Allergy Services. Clinical Commissioning Policy Proposition: anakinra/tocilizumab for the treatment adult onset Still's disease refractory to secondline therapy (adults). London: NHS England; TBC. Available from: https://www.engage.england.nhs.uk/consultation/com-policy-anakinra-tocilizumab-aosd/supporting_documents/1609aosdpolicyproposition.pdf.

3. NHS England. Clinical commissioning policy: Canakinumab for patients with Still's disease refractory to anakinra and tocilizumab (adults and children 2 years and over). Redditch: 2022. Available from: https://www.england.nhs.uk/publication/clinical-commissioning-policy-canakinumab/.

4. European Medicines Agency (EMA). Ilaris. Amsterdam: EMA; 2021. [cited 27 July 2022]. Available from: https://www.ema.europa.eu/en/medicines/human/EPAR/ilaris#:~:text=The%20active%20substance%20in%20Ilaris,body%20called%20interleukin%E2%80%911%20beta.

5. European Medicines Agency (EMA). Kineret. Amsterdam: EMA; 2022. [cited 27 July 2022]. Available from: https://www.ema.europa.eu/en/medicines/human/EPAR/kineret.

6. Food and Drug Administration. Actemra prescribing information. Silver Spring, Maryland: 2021. Available from: https://www.accessdata.fda.gov/drugsatfda_docs/label/2021/125472s044lbl.pdf.

7. Datapharm. RoActemra 162 mg solution for injection in pre-filled syringe. Summary of product characteristics. Surrey, UK: Electronic Medicines Compendium; 2021. [cited 24 February 2022]. Available from: https://www.medicines.org.uk/emc/product/5357/smpc.

5 Generalizability of SIJA to AOSD: Further Details

Some potential areas of caution when generalising from SJIA were proposed, including differences in biology between children and adults and how outcomes may differ; and variation in lifestyle habits, e.g. smoking habits in adults, which can impact on disease management. The age of AOSD onset may be another important factor because there are likely to be more differences between SJIA and AOSD in those with late-disease onset (i.e. >50 years) compared with early-disease onset (i.e. >16 years). Treatment compliance may also differ between SJIA and AOSD. Although compliance may be lower in SJIA, parents are likely to advise the clinician if this is an issue (unlike in AOSD where the patient is less likely to mention compliance issues to the clinician). However, further data are needed to support this.

A couple of the interviewed clinicians suggested that AOSD might be harder to diagnose than SJIA, and there may be differences in the diagnostic criteria used and the clinical presentation.

"[SJIA and AOSD] are two diseases both with fever and skin rash, but the rate of arthritis and systemic complications is different. The rate of comorbidities is higher in adults compared with children." **Italian clinician**

The most common learning from SJIA offered by the interviewed clinicians was the early use of biologic disease modifying anti rheumatic drugs (bDMARDs). Other suggestions were the approach to treating MAS, use of IL-6 inhibitors, ideas around genetic testing (e.g. for fever mutations), and the treat-to-target strategy used in SJIA.

6 Completed Standards for Reporting Qualitative Research (SRQR) Checklist

http://www. equator-network. org/reporting-guidelines/srqr/

**Standards for Reporting Qualitative Research (SRQR)***	**Section**
**Title and abstract**	
**Title** - Concise description of the nature and topic of the study Identifying the study as qualitative or indicating the approach (e.g., ethnography, grounded theory) or data collection methods (e.g., interview, focus group) is recommended	Title - page 1
**Abstract** - Summary of key elements of the study using the abstract format of the intended publication; typically includes background, purpose, methods, results, and conclusions	Abstract - page 3
**Introduction**	
**Problem formulation** - Description and significance of the problem/phenomenon studied; review of relevant theory and empirical work; problem statement	Section 1
**Purpose or research questio**n - Purpose of the study and specific objectives or questions	Section 1
**Methods**	
**Qualitative approach and research paradigm** - Qualitative approach (e.g., ethnography, grounded theory, case study, phenomenology, narrative research) and guiding theory if appropriate; identifying the research paradigm (e.g., postpositivist, constructivist/ interpretivist) is also recommended; rationale**	Section 2.1
**Researcher characteristics and reflexivity -** Researchers' characteristics that may influence the research, including personal attributes, qualifications/experience, relationship with	Section 2.4. Supplementary Section 1

**Standards for Reporting Qualitative Research (SRQR)***	**Section**
participants, assumptions, and/or presuppositions; potential or actual interaction between researchers' characteristics and the research questions, approach, methods, results, and/or transferability	
**Context** - Setting/site and salient contextual factors; rationale**	Sections 2.2 & 2.3
**Sampling strategy** - How and why research participants, documents, or events were selected; criteria for deciding when no further sampling was necessary (e.g., sampling saturation); rationale**	Section 2.2. Supplementary Section 1
**Ethical issues pertaining to human subjects** - Documentation of approval by an appropriate ethics review board and participant consent, or explanation for lack thereof; other confidentiality and data security issues	Sections 2.1 & 2.2. Supplementary Section 1
**Data collection methods** - Types of data collected; details of data collection procedures including (as appropriate) start and stop dates of data collection and analysis, iterative process, triangulation of sources/methods, and modification of procedures in response to evolving study findings; rationale**	Sections 2.1, 2.4 & 2.5
**Data collection instruments and technologies** - Description of instruments (e.g., interview guides, questionnaires) and devices (e.g., audio recorders) used for data collection; if/how the instrument(s) changed over the course of the study	Sections 2.3, 2.4 & 2.5.
**Units of study** - Number and relevant characteristics of participants, documents, or events included in the study; level of participation (could be reported in results)	Section 3 & Supplementary Table 1

**Standards for Reporting Qualitative Research (SRQR)***	**Section**
**Data processing** - Methods for processing data prior to and during analysis, including transcription, data entry, data management and security, verification of data integrity, data coding, and anonymization/de-identification of excerpts	Sections 2.1, 2.4 & 2.5. Supplementary Section 1
**Data analysis** - Process by which inferences, themes, etc. , were identified and developed, including the researchers involved in data analysis; usually references a specific paradigm or approach; rationale**	Section 2.5
**Techniques to enhance trustworthiness** - Techniques to enhance trustworthiness and credibility of data analysis (e.g., member checking, audit trail, triangulation); rationale**	Section 2.5
**Results/findings**	
**Synthesis and interpretation** - Main findings (e.g., interpretations, inferences, and themes); might include development of a theory or model, or integration with prior research or theory	Sections 3, 4.1 & 4.2
**Links to empirical data** - Evidence (e.g., quotes, field notes, text excerpts, photographs) to substantiate analytic findings	Section 3
**Discussion**	
**Integration with prior work, implications, transferability, and contribution(s) to the field -** Short summary of main findings; explanation of how findings and conclusions connect to, support, elaborate on, or challenge conclusions of earlier scholarship; discussion of scope of application/generalizability; identification of unique contribution(s) to scholarship in a discipline or field	Sections 3 & 4

**Standards for Reporting Qualitative Research (SRQR)***	**Section**
**Limitations** - Trustworthiness and limitations of findings	Section 3.1
**Other**	
**Conflicts of interest** - Potential sources of influence or perceived influence on study conduct and conclusions; how these were managed	Declarations of Interest section
**Funding** - Sources of funding and other support; role of funders in data collection, interpretation, and reporting	Funding section

* The authors created the SRQR by searching the literature to identify guidelines, reporting standards, and critical appraisal criteria for qualitative research; reviewing the reference lists of retrieved sources; and contacting experts to gain feedback. The SRQR aims to improve the transparency of all aspects of qualitative research by providing clear standards for reporting qualitative research.

** The rationale should briefly discuss the justification for choosing that theory, approach, method, or technique rather than other options available, the assumptions and limitations implicit in those choices, and how those choices influence study conclusions and transferability. As appropriate, the rationale for several items might be discussed together.

Reference: O'Brien BC, Harris IB, Beckman TJ, Reed DA, Cook DA. Standards for reporting qualitative research: a synthesis of recommendations. Academic Medicine, Vol. 89, No. 9 / Sept 2014

DOI: 10. 1097/ACM. 0000000000000388

7 Supplementary Tables

Supplementary Table 1: Overview of interviewee characteristics.

**Country**	**Role**	**Experience (years)**
**UK**	Clinical academic / director of clinical immunology and allergy	15
	Rheumatology consultant	7
	Academic rheumatologist	28
**Italy**	Doctor in rheumatology and clinical immunology / head of autoinflammatory disease clinic	7
	Rheumatologist and clinical immunologist	6
	Assistant professor of rheumatology	10
	Rheumatologist	4 to 5
**France**	Rheumatologist	6
	Internist (specialised in clinical immunology)	>10
**Germany**	Senior consultant (internal medicine and rheumatology) / head of clinical trial unit	>15
	Rheumatologist	27

Key: UK – United Kingdom.

Supplementary Table 2: Variation in dose across treatments.

Corticosteroids	csDMARDs	Biologic disease modifying anti rheumatic drugs (bDMARDs)*
**UK**		
		Anakinra: daily injections or 100mg/day or two/three injections per day if needed (approximately 25% of patients need a double dose).
	Methotrexate: 15mg or 20mg/week.<br><br>Cyclosporine: 100mg to 150mg.	Anakinra: 100mg to 200mg/day. If MAS in hospital up to 1,000mg/day.<br><br>Tocilizumab: 162mg/week.<br><br>IV Tocilizumab: 8mg/kg on a four-weekly basis.
Oral steroids: 30mg to 60mg/day.<br><br>IV steroids: 3g in 3 days if the patient has a severe presentation with MAS.	Methotrexate: 25mg/week.<br><br>Cyclosporine: 3mg to 4mg/kg (for six months maximum before discontinuation).	Anakinra: daily injections or 100mg/day and 300mg/day if severe.
**Italy**		
Low/medium dose for most patients, but if the patient has MAS, a very high dose of IV glucocorticoids.		Anakinra: daily injections or 100mg/day.<br><br>Canakinumab: adjusted to body weight, start with the conventional dose (4mg/kg) and increase if needed.
1mg/kg if there are no visceral manifestations or 500mg to 1,000mg daily if there are visceral manifestations.	Methotrexate: 10mg to 15mg/week increasing to 20mg to 25mg/week.<br><br>Cyclosporine: initially 3mg/kg initially increasing to 5mg/kg if needed.	Anakinra: up to four/five injections/day if needed.<br><br>Canakinumab: double dose from 150mg to 300mg can be used.

**Corticosteroids**	**csDMARDs**	**Biologic disease modifying anti rheumatic drugs (bDMARDs)***
0.5 to 1mg/kg for non-complicated AOSD.	Methotrexate: 7.5mg to 15mg/week.	Anakinra: 100mg to 200mg/day.
**France**		
	Methotrexate: 10mg to 25mg/week.	Anakinra: daily injections or 100mg/day.
**Germany**		
	Methotrexate: 20mg/week if a senior clinician is prescribing. 10 to 15mg/week if a junior clinician is prescribing.	
		Anakinra: 100mg to 200mg/day.

Key: AOSD - adult-onset Still's disease; csDMARD - conventional synthetic disease-modifying antirheumatic drug; IV – intravenous; MAS – macrophage activation syndrome; UK – United Kingdom

* Canakinumab is not currently used routinely in the UK for reimbursement reasons.

Supplementary Table 3: Important outcomes, clinical measures of severity (systemic score) and patient reported outcome measures.

**Other efficacy and effectiveness outcomes offered**	**Other clinically relevant treatment-related adverse events suggested**	**Alternative AOSD-specific patient-reported outcome (PRO) measures**
Changes in clinical characteristics (e.g. fever, rash, fatigue, joint control, pain).<br><br>Changes in laboratory characteristics (e.g. CRP, ferritin, ESR, full blood chemistry).<br><br>Number of flares over time.<br><br>Proportion of patients experiencing relapse-free survival.<br><br>Rates of disability.<br><br>Articular destruction, joint erosion over time, need for joint/hip replacement, and structural damage on joint imaging.<br><br>Changes in clinical scores (systemic score, Disease Activity Score 28 joints (DAS28), painful joint count, Health Assessment Questionnaire (HAQ), PROs around daily functioning).<br><br>Time to return to work.<br><br>Time from symptom onset to diagnosis.	Liver dysfunction.<br><br>Cancer risk.<br><br>Cancer incidence/diagnosis.<br><br>Liver toxicity (due to methotrexate).<br><br>Sepsis.<br><br>Organ function.<br><br>Hospital admission.<br><br>Drug tolerability.	Fatigue Assessment Scale (FAS).<br><br>Beck Depression Inventory (BDI).<br><br>Work Ability Score (WAS).<br><br>Auto-Inflammatory Diseases Activity Index (AIDAI)*.<br><br>Glucocorticoid Toxicity Index (GTI).<br><br>Physician and patient global assessment score.<br><br>Hanover Functional Ability Questionnaire (FFbH).<br><br>DAS28.

* Piram M, Kone-Paut I, Lachmann HJ, Frenkel J, Ozen S, Kuemmerle-Deschner J, et al. Validation of the auto-inflammatory diseases activity index (AIDAI) for hereditary recurrent fever syndromes. Ann Rheum Dis. 2014.73(12):2168-73. doi: 10.1136/annrheumdis-2013-203666.
